# *In-situ* FTIR spectroscopy of epoxy resin degradation: kinetics and mechanisms

**DOI:** 10.3389/fchem.2024.1476965

**Published:** 2024-10-30

**Authors:** Marianna Pannico, Giuseppe Mensitieri, Pellegrino Musto

**Affiliations:** ^1^ Institute of Polymers, Composites and Biomaterials, National Research Council of Italy, Pozzuoli, Italy; ^2^ Department of Chemical, Materials and Production Engineering, University of Naples Federico II, Naples, Italy

**Keywords:** FTIR, epoxy, degradation, kinetics, DFT, mechanism

## Abstract

We report on an *in situ* FTIR study of the thermo-oxidative degradation of a flexible epoxy resin. Different and complementary approaches to the analysis of the spectral data were employed, providing a detailed description of the process in terms of kinetics and mechanisms. A preliminary normal coordinate analysis, based on the DFT method, allowed for a reliable interpretation of the observed spectrum, increasing the amount of available structural information. Two-dimensional correlation spectroscopy provided details on the evolution of the reacting network structure. The relative stability of the various functional groups was ranked, and the most likely sites of initiation were identified. Oxygen fixation on the network chains produced amide and ketone groups, with the latter developing at a higher rate. The kinetic profiles of various functional groups were accurately simulated by a first-order, biexponential model, which allowed a quantitative comparison among their relative stabilities. The spectroscopic analysis allowed us to propose likely mechanisms and to identify those that occur preferentially.

## 1 Introduction

Epoxy resins find application in a number of technological sectors owing to their excellent properties and versatility. They represent the main class of matrices for structural composites and are widely used as adhesives, surface coatings, and laminates. Their cured networks exhibit high strength, low creep, negligible cure shrinkage, excellent resistance to corrosion, and reasonable thermal stability (Ratna, 2009; Pascault et al., 2013; Ashcroft et al., 1993). The versatility of epoxy resins stems from the wide choice of available amine hardeners which can be aliphatic or aromatic and give rise to materials with properties ranging from those of rubber to those of a very rigid and brittle network, with glass transition temperatures (T_g_’s) of 230–503 K (Ashcroft et al., 1993; Ellis and Ellis, 1993; Bonnaud et al., 2000). A recent development is their application in the field of vibrational damping (Ratna et al., 2004). Vibration is a major concern in dynamic systems as it produces several undesired effects, including noise, fatigue, degraded performance, and the eventual failure of the structure (Barber, 2002; Jones, 2001). Vibration control may be achieved by coupling a viscoelastic material (typically a polymer) with the vibrating surface. The vibrational energy is thus converted into heat and dissipated by the damping polymer (Murayama, 1978; Ferry, 1980). The formulation presented in this study was specifically designed for this purpose, exploiting the viscoelastic behaviour of the network, coupled with its excellent adhesive properties. A mixture of short- and long-chain aliphatic polyetheramines (jeffamines) has been used as the curing blend, resulting in a rubbery network with a T_g_ of −33 °C. In this application, a main requirement is thermooxidative stability; in fact, continuous mechanical cycling usually induces large temperature excursions, which become more pronounced when the efficiency of the dynamic structure degrades. In general, the thermal stability of epoxies is a major factor affecting their shelf life and the maximum service temperature for any kind of application. A large body of research already exists on the thermo-oxidative degradation of epoxy resin, mainly concerned with the rigid networks produced by aromatic curing agents (Lee et al., 2001; Musto et al., 2001; Lee, 1965a; Rose et al., 1993; Li et al., 2021; Zhou et al., 2020). Much less information is available on systems cured with aliphatic diamines (Lee et al., 2001; Ratna et al., 2024; Xia et al., 2014; Mailhot et al., 2005a; Krauklis and Echtermeyer, 2018) and, in particular, with flexible, high molecular mass polyetheramines (Ratna et al., 2004; Ratna et al., 2024; Mailhot et al., 2005a; Krauklis and Echtermeyer, 2018). Among the available research literature, a noteworthy contribution (Krauklis and Echtermeyer, 2018) investigated the hygrothermal degradation of a flexible epoxy network employing a combination of complementary characterization techniques, including FT-NIR, ATR-FTIR, energy-dispersive X-ray analysis (EDX), high-resolution–inductively coupled plasma–mass spectrometry (HR-ICP-MS), optical microscopy, scanning electron microscopy (SEM), and dynamic mechanical thermal analysis (DMTA). It was concluded that in the investigated aging conditions (water immersion, 60 °C), chain-scission does not occur, and the oxidative process only produces in-chain carbonyls. No amide terminals were detected nor a depletion of aromatic moieties. Conversely, in more drastic conditions (UV irradiation and high-T exposure), different spectral features and alternative degradation pathways were detected (Mailhot et al., 2005a; Mailhot et al., 2005b), prompting us to reconsider system stability under specific service conditions by using a different experimental approach which emphasizes the kinetics of the process. The degradation of solids is customarily investigated by thermogravimetric analysis (TGA) in the isothermal or non-isothermal mode (Vyazovkin and Linert, 1994; Ozawa, 1965; Doyle, 1962). Other techniques are frequently used to complement TGA, among which are gas chromatography (Bishop and Smith, 1970), mass spectroscopy (Lee, 1965b), radiochemical analysis (Bishop and Smith, 1970), and combinations of these. A common issue of the above experimental approaches is that they rely on volatile products generated upon thermo-oxidation. Reaching a detectable amount of such volatiles requires very high degradation temperatures, which may cause intricate processes, such as the rearrangement of degradation fragments and further decomposition of primary products. Thus, analysis of volatile products provides relevant information on temperature stability but is hard to interpret mechanistically. These issues are alleviated by the use of a solid-state spectroscopic approach such as Fourier transform infrared (FTIR). In this case, the technique considers the evolution of the network rather than the evolved volatiles; due to its sensitivity, it is able to detect weak features occurring in the early stages of the process, related to the initiation step(s) (Musto et al., 2001; Mailhot et al., 2000; Koenig, 1975). We adopted transmission sampling in place of the more widespread attenuated total reflection mode (ATR) because it allows full contact of the sample surfaces with the environment and facilitates *in situ*, time-resolved data collection. Furthermore, the transmission mode is the best choice for an accurate and reproducible quantitative analysis (Chalmers and Griffiths, 2002). Clean kinetic data can thus be gathered, allowing us to compare the chemical stability of the different functional groups of the network. Large collections of dynamically changing datasets also permit advanced spectral-analysis approaches such as two-dimensional correlation spectroscopy (2D-COS), least squares curve fitting (LSCF) analysis, and difference spectroscopy (DS). Among other advantages, such tools are able to improve the band-shape resolution, which is frequently poor in systems undergoing photo- or thermo-oxidative processes (Musto et al., 2008; Musto et al., 2012; Musto, 2003). QM-based normal coordinate analysis (QM-NCA) is the tool for a quantitative interpretation of the spectral features, making it possible to extract the maximum amount of information at the molecular level from the vibrational pattern. The kinetic data coupled with the structural insights provided by spectroscopic analysis allowed us to propose likely mechanisms for the degradation process. A further scope of the present contribution is to highlight the potential of FTIR spectroscopy, in connection with its advanced interpretative tools, for the molecular characterization of complex transient phenomena in polymeric materials.

## 2 Materials and methods

### 2.1 Materials

Diglycidyl ether of bisphenol-A (DGEBA) M.W. = 355 Da, polyetheramine (jeffamine) D230 M.W. = 230 Da, and polyetheramine D2000 M.W. 2000 Da were purchased from Sigma-Aldrich, Italy. These products were used as received, with no further purification.

The structural formulae of the reagents are reported below:

DGEBA 
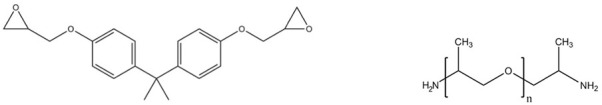


jeffamines D230, D2000 

### 2.2 Preparation protocol

The epoxy network to be investigated was obtained by curing the DGEBA precursor with an equimolar mixture of D230 and D2000 jeffamines. We transferred 6.00 g (16.9 mmol) of DGEBA, 0.972 g (4.226 mmol) of D200, and 8.452 g (4.226 mmol) of D2000 were to a round flask and kept this under mechanical stirring at 60 °C until complete homogenisation. The viscous, transparent mixture thus obtained was degassed under vacuum until air bubbles completely disappeared (30 min), and it was cooled to RT. Two glass plates were treated with a releasing agent (Lubrolene E6) and preheated at 60 °C. A small amount of the resin precursor was squeezed between the hot plates and gently pressed at 5.0 bar to obtain a film thickness suitable for transmission IR measurements. The film was cured at 80 °C for 2 h and post-cured at 140 °C for 6 h. All thermal treatments were performed under an N_2_ atmosphere to prevent premature oxidation. After cooling to RT, the mould was opened, and a film 8 ± 2 μm thick was recovered. It was rinsed with dichloromethane to eliminate residual releasing agent from the surfaces and dried in air prior to introduction into the environmental chamber of the spectrometer. The *T*_*g*_ of the fully cured resin as detected by DSC was −33 °C.

### 2.3 FTIR spectroscopy

The isothermal collection of transmission FTIR spectra was performed *in situ*, in the *time-resolved* mode. The measurements were made under gas flow (50 cm^3^ s^-1^) using a modified Linkam cell, THMS350V (Surrey, UK), equipped with temperature control (−180 ÷ 350°C) and a vacuum system. The cell was operated through service lines connected to mass flow controllers (MKS Type GM50A (Andover, MA)) for setting the gas flux, while a solenoid valve regulated the pressure inside the cell. The system was equipped with a Pirani vacuometer and a MKS Baratron 121 pressure transducer (produced by MKS Instruments, Andover, MA, full-scale 1,000 Torr, resolution 0.01 Torr, accuracy equal to ±0.5% of the reading). The environmental chamber was fluxed with dry N_2_ up to stabilization at the test temperature (180 °C); thereafter, the flowing gas was switched to air. The switching time marks the origin of the kinetic time-scale. During the interval to stabilize the test temperature (≈15 min), no modifications of the IR spectrum were observed. Prolonged exposure (1 h) at 180 °C under nitrogen confirmed the stability of the spectrum in the absence of oxygen.

**SCHEME 1:**
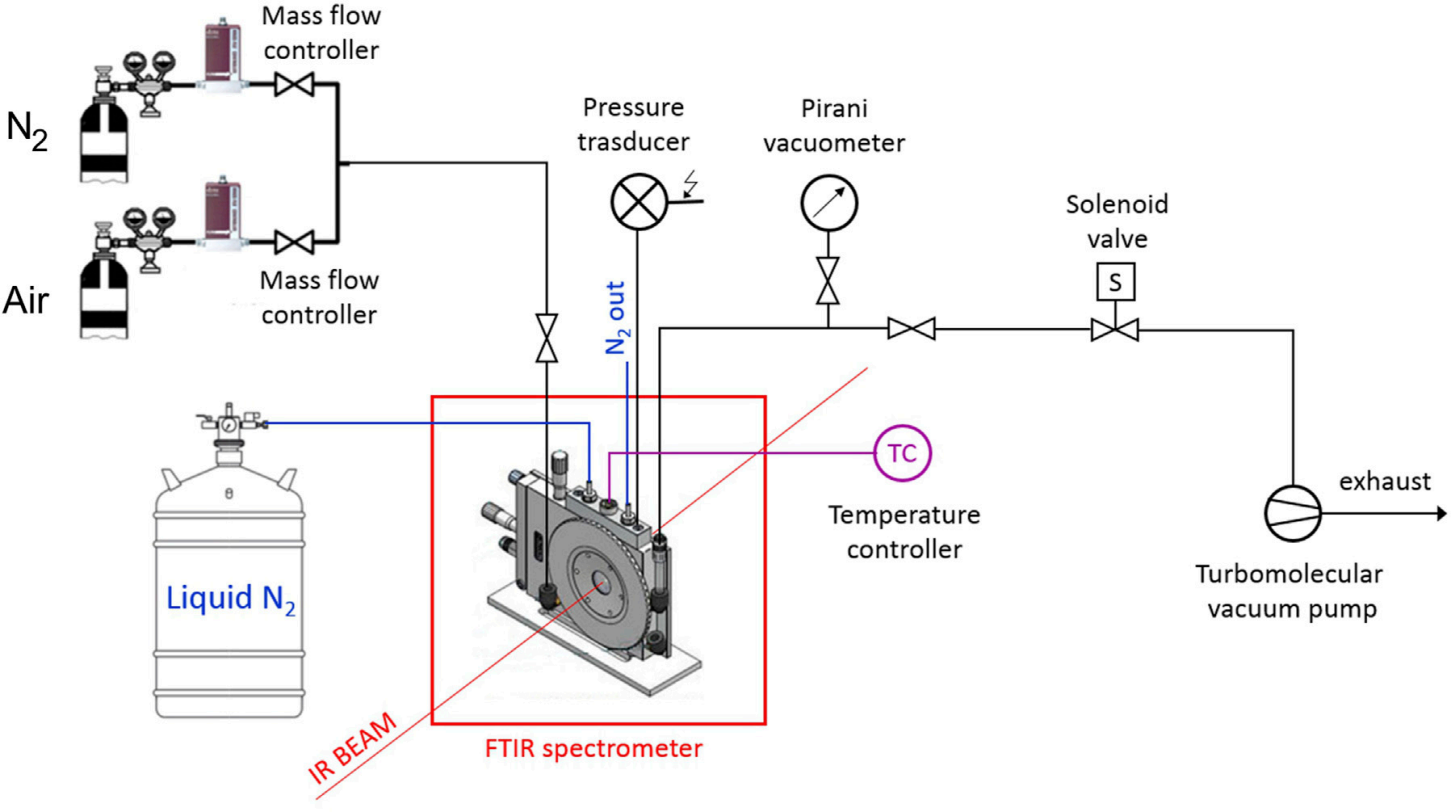
Experimental setup for the collection of FTIR spectra.

The FTIR instrument was a Spectrum-100 spectrometer (Perkin-Elmer, Norwalk, CT, United States) equipped with a Ge on KBr beam splitter and a wide-band DTGS detector. Instrumental parameters were set as follows: resolution, 2 cm^-1^; optical path difference (OPD) velocity, 0.20 cm/s; frequency range, 4,000–450 cm^-1^. A schematic diagram of the experimental apparatus is reported in Scheme 1 .

### 2.4 Computational details

A preliminary exploration of the potential energy surface of the model compound was made by Molecular Mechanics (MM) using the MMFF94 force field (Merck Molecular Force Field, from Merck Co, Kenilworth, NJ, United States) (Halgren, 1996). A Metropolis–Monte Carlo method (Rappè and Casewit, 1997) was adopted for the systematic variation of multiple dihedral angles. Four conformational degrees of freedom were considered (Supplementary Figure S1, Supplementary Material—SM). Quantum mechanical (QM) calculations were performed on the minima identified by the MM conformational search using density functional theory (DFT) based on linear combination of atomic orbitals (LCAO) with the unrestricted spin polarization method. The adopted Hamiltonian was the B3LYP global hybrid functional (Becke, 1992; Lee et al., 1988) as implemented in the Gaussian16 program package (Frisch et al., 2016) (Gaussian Inc., Pittsburgh, PA) in connection with the standard 3-21G basis set. Larger basis sets were also checked and were found to provide limited improvements in the simulation of the vibrational spectrum. After geometry optimization, a normal coordinate analysis at the same level of theory was performed, comprising the calculation of the Hessian matrix (**F**) by analytical evaluation of the first and second derivatives of the potential energy with respect to the Cartesian displacement coordinates. The **F** matrix was then transformed in terms of mass-weighed coordinates and diagonalized to obtain the corresponding eigenvalues (normal frequencies) and eigenvectors (displacement vectors, **L** matrix). Finally, a transformation into a set of non-redundant internal coordinates of both the **F** and **L** matrices was accomplished in order to characterize the normal modes in terms of their potential energy distribution (PED), expressed, in normalized form, as Wilson et al. (1955).

$${\left(PED\right)}_{jk}=\frac{F_{jj}L_{jk}^{2}}{\sum_{i}F_{ii}L_{ik}^{2}}\cdot 100.$$

PED % refers to the contribution of the *j* ^th^ internal coordinate to the *k* ^th^ normal mode, *F*_*jj*_ is the *j* ^th^ diagonal force constant, and *L*_*jk*_ is the corresponding element of the **L** matrix.

## 3 Results and discussion

### 3.1 QM-based normal coordinate analysis

**FIGURE 1:**
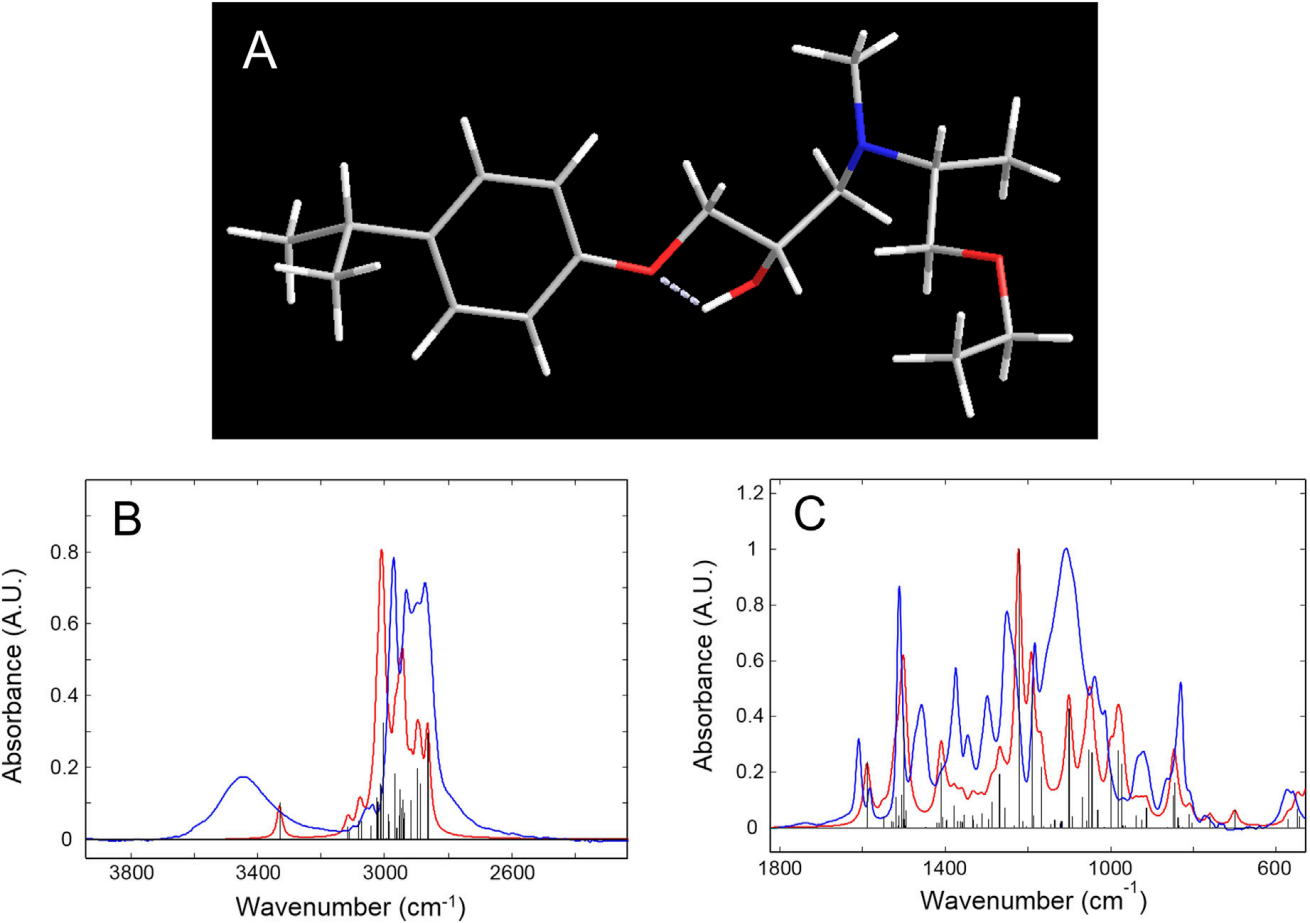
**(A)**: Geometry of the model compound optimized at the B3LYP/3-21G level of theory. **(B, C)** Blue trace: experimental spectrum of the epoxy network. Red trace: calculated spectrum of the network’s model compound with a Lorentz broadening function. Black bar graph: calculated spectrum without broadening. All spectra are normalized with respect to their maximum absorbance values.

**FIGURE 2:**
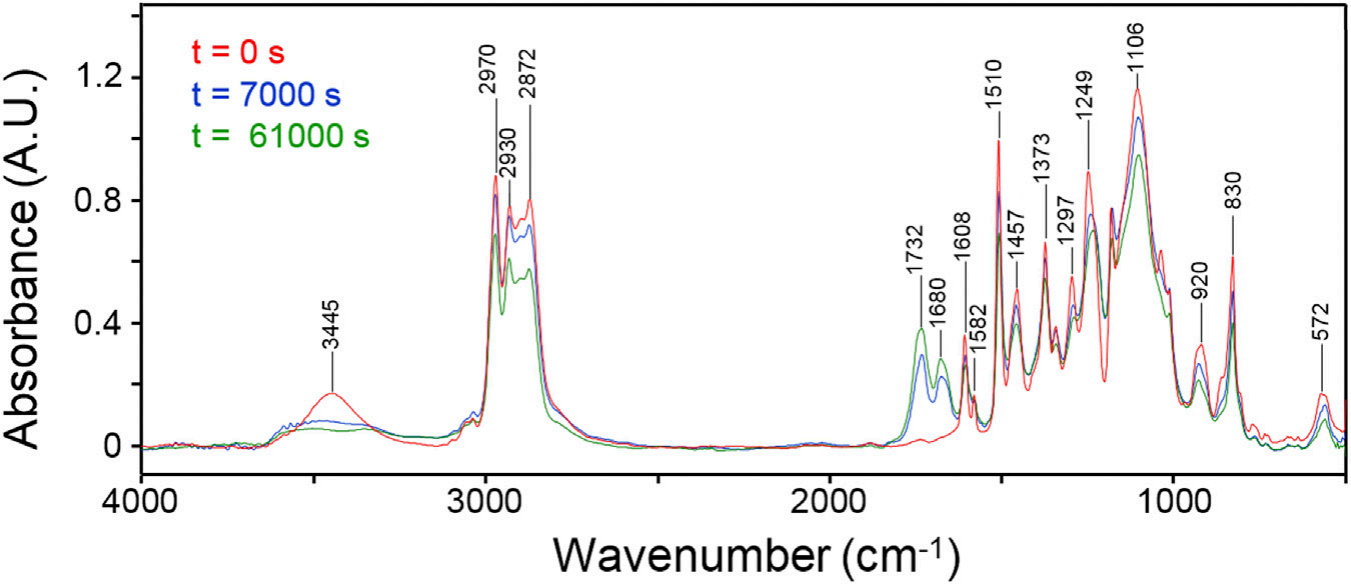
FTIR spectra of the network collected at different times during the thermo-degradation experiment. Collection times as indicated.

The as-collected IR spectrum of the epoxy network is reported in Figure 2 (red trace) and Figure 1 (blue trace) after normalization with respect to the maximum absorbance value (Figure 1B refers to the frequency range 3,900–2,200 cm^-1^; Figure 1C to the 1800–550 cm^-1^ range).

The pattern displays numerous features, providing abundant structural information. The specific sample preparation has ensured that the whole spectrum lies well within the absorbance-concentration linearity range (less than 1.5 absorbance units) which allows for the use any peak for quantitative analysis. The amount of available molecular information depends on the interpretative level, ideally providing a normal mode description to any observed feature of the spectrum. So far, despite the relevance of epoxy resins and the widespread use of vibrational spectroscopies for their characterization, only qualitative assignments are available, based on correlative approaches (Musto et al., 2001; Ratna et al., 2024; Ngono et al., 1999; Ngono and Maréchal, 2000; Rivaton et al., 1997a). Furthermore, in the main reference papers (Ngono et al., 1999; Zhang et al., 1994; Mertzel and Koenig, 2005), conflicting assignments are often reported, even for the major peaks, which prompted us to adopt a more rigorous approach based on the currently available QM methods.

The choice of the model compound was made by considering the network structure depicted in Supplementary Figure S2, SM. A molecular fragment representative of the polymer chains forming the 3D network was identified, containing all the relevant functional groups. It was truncated by a H atom on the isopropylidene site and the tertiary amine group, and with a methyl group on the ether terminal. Owing to the complexity of the molecular structure, a preliminary survey of the potential energy surface was accomplished by a Metropolis–Monte Carlo method (Rappè and Casewit, 1997) for the systematic variation of multiple dihedral angles; the *τ*_*1*_ *, τ*_*2*_ *, τ*_*3*_, and *τ*_*4*_ torsions were considered (Supplementary Figure S1, SM). Then, the most promising structures were fully optimized at the B3LYP/3-21G level of theory, leading in all cases to the relaxed structure represented in Figure 1A and in Supplementary Figure S1, SM, with the relative atom numbering. Normal coordinate analysis (NCA) according to the Wilson GF method (Wilson et al., 1955) was performed next, whose results are compared to the experimental values in Figures 1B and C. Table 1 summarizes the NCA, also reporting the normal-mode description of the observed peaks, along with their potential energy distribution (PED).

**TABLE 1:** Experimental and calculated frequencies, percent error, potential energy distribution, and description of the vibrational modes in the spectrum of the investigated epoxy network.

Exp (cm^-1^)	Calc (cm^-1^)	Err (%)	PED (%)	Description
-	3,331 (w)	–	s1 (100)	ν(OH)
3,097 (w)	3,116 (w)	0.6	s4 (95), s3 (90)	ν(CH)_Ar_
3,057 (w)	3,076 (w)	0.6	s2 (91), s5 (96)	ν(CH)_Ar_
2,970 (s)	3,004 (s)	1.3	s8 (78), s23 (83), s20 (77) s23 (11)	ν_asym_ (CH)_3_/ν_asym_ (CH)_2_ (α to N)
2,930 (s)	2,945 (m)	0.5	s17 (12) s31 (78), s9 (84)	ν_sym_ (CH)_3_ (all)
2,895 (s)	2,895 (m)	0	s28 (85), s26 (75) s28 (11)	ν(CH)_2_ (α to O)
2,872 (s)	2,865 (m)	0.2	s24 (94)	ν(CH) (α to N)
		$\bar{Err}$ = 0.5 ±0.44		
1,608 (m)	1,588 (m)	1.2	s33 (52) s57 (16) s87 (11)	in-plane ring def/*ν*(CO)
1,582 (m)	1,550 (w)	2.0	s32 (50) s59 (16)	in-plane ring def
1,510 (vs.)	1,500 (s)	0.7	s55 (53)	sc (CH_2_) α to O
1,457 (m)	–	–		–
1,373 (m)	1,408 (m)	2.5	s54 (31) s78 (10) s147 (13)	δ(COH)/δ(CCH)
1,343 (m)	1,360 (w)	1.3	s80 (15) s128 (19) s130 (24)	δ(CCH) (next to N)
1,297 (m)	1,267 (m)	2.3	s51 (57)	in-plane ring def [ν(CC)]
1,249 (s)	1,221 (vs.)	2.2	s36 (46) s57 (11)	ν(O–Ar)
1,183 (m)	1,190 (m)	0.6	s33 (13) s57 (40)	δ(CCH)_Ar_
1,153 (sh, m)	1,167 (m)	1.2	s66 (10)	skeletal (R)
1,106 (vs.)	1,100 (m), 1,070, 1,052	0.5	s35 (27) s42 (16), s49 (13) s132 (16) s40 (26) s125 (16)	ν(O–CH_2_)/δ(CH_3_)
1,038 (m)	1,046 (m)	0.6	s42 (23) s44 (13) s116 (13)	*ν*(C–N)/*tw* (CH_2_)
1,013 (m)	1,000 (m)	1.3	s39 (12) s41 (12)	*δ*(COH)/*ν*(C–N)
934 (m)	938 (w)	0.4	s43 (35) s50 (15) s52 (13)	*ν*(O–CC_2_H_5_)
920 (m)	926, 913 (w)	0.05	s51 (12) s118 (14)	*tw* (CH_2_)
862 (sh)	850 (sh)	1.4	s46 (50)	–
830 (m)	845 (m)	2.0	s107 (64)	*w* (CCH)_Ar_
805 (w)	809, 805 (w)	0.2	s35 (27) s37 (15)	in-plane ring def [ν(CC)]
773 (vw)	760 (w)	1.7	s45 43	*ν*_*sym*_ (CN)
572 (w)	570 (m)	0.3	s85 18 s86 43 s87 12	skeletal (L)
559 (w)	550, 543 (w)	2.2	s106 12 s152 18 s153 -24 s92 -15 s101 -18	*w* (CCH)_Ar_/*w* (COH)
		$\bar{Err}$ = 1.2 ± 0.78		

$Err\;\%=\frac{\left|\nu_{\exp}-\nu_{calc}\right|}{\nu_{\exp}}\times 100;$ ν = stretching; δ = bending; sc = scissoring; tw = twisting; w = wagging; Ar = aromatic. Figures in round brackets in column 4 (PED) are the % contributions of the internal coordinates to the total PE. Contributions lower than 10% were neglected. Contributions separated by commas indicate the occurrence of unresolved normal modes at the observed frequency. R and L in column 5 (Description) denote right and left side of the molecular model as indicated in Supplementary Figure S1, SM.

The correlation between the calculated and experimental frequencies is reported in Supplementary Figure S3, SM. The linearity with zero intercept extends over the whole range (*R* ^*2*^ = 0.9995), and the slope, representing the correction factor to compensate for neglecting the exact treatment of the electron correlation, is 0.963 ± 0.004. This value is in excellent agreement with the benchmark value of 0.965 reported in the literature (Kesharwani et al., 2015; Precomputed vibrational scaling factors, 2019). This analysis confirms the soundness of the NCA and of the proposed assignments and shows that anharmonicity, which produces non-linearity effects in the high-frequency side (CH/OH stretchings), is negligible in the present case.

In the high-frequency side, the calculated ν(OH) vibration occurs as a sharp peak at 3,331 cm^-1^, while the observed spectrum displays a broad absorption centered at 3,445 cm^-1^. The lack of correlation in this region is due to incomplete molecular modeling; the chain fragment is considered to be isolated *in vacuo* and does not take into account intermolecular H-bonding interactions with neighboring chains that are responsible, in the actual network, for the broadening of the observed ν(OH) band. In the model, only an intramolecular H-bonding with the ether oxygen (O_10_) is formed, having a conspicuous interaction energy, as demonstrated by the position of the relative normal mode. The ν(CH) range between 3,250 and 2,800 cm^-1^ is reproduced with remarkable accuracy by the adopted model chemistry, with an average error of 0.5%, well within the predictive capabilities of the method (≈2%) (Kesharwani et al., 2015); this affords the interpretation of the complex profile in this interval. In particular, as well as the aromatic signals above 3,000 cm^-1^, the two components at 2,970 and 2,930 cm^-1^ are due to the methyl groups, with the latter mainly localized on the isopropyl terminal. The 2,895 and 2,865 cm^-1^ peaks originate, respectively, from the CH_2_ in α to the ether oxygen (C_21_) and the CH in α to the nitrogen (C_18_–H_46_). These assignments afford a signature of specific molecular sites to monitor the initiation and advancement of the degradation process.

In the range below 1,800 cm^-1^ the experimental-to-calculated correlation worsens slightly (average error = 1.2%), as generally occurs when there is a significant degree of mixing among the basic vibrations (Wilson et al., 1955). However, considering both the positions and the intensities of the simulated spectrum, a reliable description of the observed features can be achieved. The doublet at 1,608–1,582 cm^-1^ is due to the aromatic ring with a minor contribution from the C_4_–O_10_ stretch. The prominent peak at 1,510 cm^-1^ is mainly due to the CH_2_ scissoring at C_11_, while the equally intense feature at 1,249 cm^-1^ is a mixed vibration with a prevailing O–Ar character. These rather localized modes are also diagnostic for the recognition of the degradation mechanism. The most intense band in the experimental spectrum, centered at 1,106 cm^-1^, is broad and asymmetric—likely due to an unresolved multicomponent structure. The calculated spectrum in this range displays lesser intensity and a simpler structure (three fully resolved components at 1,100, 1,070 and 1,052 cm^-1^). These modes are extensively mixed (see PED in Table 1) and are of limited utility in the present context. In addition, of interest for the spectroscopic analysis are the 1,183 cm^-1^ peak (aromatic) and the partially resolved doublet at 862–830 cm^-1^ assigned, respectively, to a skeletal mode with prevalent N_15_–C_16_ character and to a ring mode (CCH wagging). The remaining features are either too weak or too mixed to be useful for analytical purposes.

### 3.2 2D-correlation spectroscopy

Two-dimensional correlation spectroscopy (2D-COS) is a powerful tool for investigating dynamic processes that produce extensively overlapped patterns with limited or no resolution (Noda and Ozaki, 2004). It has been successfully applied to investigate thermo- and photo-oxidative degradation kinetics (Musto et al., 2008; Musto et al., 2012; Musto, 2003). The method, rooted in the principles of time-series analysis, improves the resolution by spreading the data over a second frequency axis; at the same time, it can characterize the dynamics of the evolving system at a finer level than conventional 1D-spectroscopy. We briefly summarize the principles of the method for convenience (Noda and Ozaki, 2004). The analysis produces two maps denoted as “synchronous” and “asynchronous”, which convey different information. Along the main diagonal (power spectrum), the synchronous spectrum highlights the signals that are more sensitive to the applied perturbation. If the response function can be expressed in the form of an exponential decay, as in the present case (*vide infra*), then the cross-correlation peaks at off-diagonal positions reflect any couple of signals undergoing intensity changes in the sampling interval. Their sign is positive if both peaks change in the same direction (they both increase or decrease) and are otherwise negative. The asynchronous spectrum displays a peak at *x-y* coordinates [ν_1_,ν_2_] when the two corresponding IR signals change at different rates and zero intensity if they change at the same rate. This effect produces the resolution enhancement and the specific shape of the asynchronous pattern. In addition, the sign of the asynchronous peaks defines the relative rate of the correlated signals according to the so-called Noda’s rules (Noda and Ozaki, 2004; Noda, 2000). The most important are the following: an asynchronous cross-peak located at coordinates [ν_1_,ν_2_] is positive if the intensity change at ν_1_ is accelerated with respect to that at ν_2_ and is negative otherwise. This remains true as long as the synchronous spectrum at [ν_1_,ν_2_] is positive; otherwise, the above relationship is to be reversed.

Figure 2 displays the initial spectrum of the network (red trace) and those collected after 7,000 and 61,000 s (blue and green traces, respectively). An intensity decrease of most peaks is observed as a consequence of functional group depletion, and the emergence of two well-resolved components in the carbonyl range (1,732–1,680 cm^-1^) assigned, respectively, to ester and amide functionalities.

**FIGURE 3:**
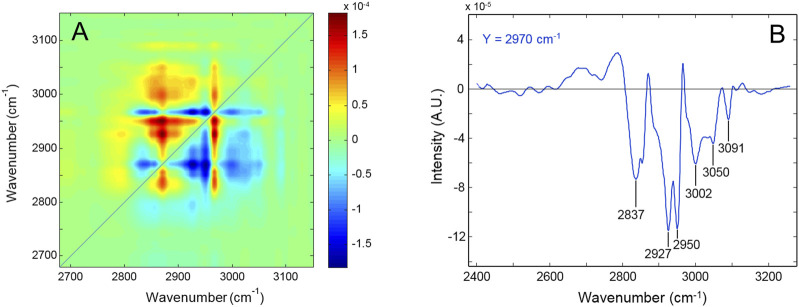
**(A)** Asynchronous spectrum in the 2,700–3,000 cm^-1^ range of the network subjected to thermo-oxidation at 180°. **(B)** Y-isofrequency plot at 2,970 cm^-1^.

Figure 3A displays the asynchronous pattern in the 2,700–3,000 cm^-1^ range obtained from the *time-resolved* spectra collected during the degradation process. The full sequence of spectra employed for the 2D-COS analysis is displayed in Supplementary Figures S4 and S5, SM. Figure 3B represents the *Y*-isofrequency line at 2,970 cm^-1^. The resolution enhancement brought about by the 2D approach is evident. The main feature is represented by the negative correlations of the peak at 2,970 cm^-1^ with the components at 2,837, 2,927, 2,950, 3,002, 3,050, and 3,091 cm^-1^ (Figure 3B). The NCA assigned the 2,970 cm^-1^ peak to unresolved CH_3_/CH_2_ stretching, which implies that the aromatic signals (3,050–3,091 cm^-1^) decrease faster than the aliphatic ones. It is noted that the number of components detected by 2D-COS largely exceeds the spectroscopic resolution and, hence, the level of detail achievable with the QM vibrational analysis. For instance, the maximum observed at 2,970 cm^-1^ is found to comprise three components at 2,838, 2,853, and 2,970 cm^-1^. A full account of the features observed in the asynchronous map is therefore unfeasible; however, relevant information on the relative rate of depletion (i.e., the relative stability) of the different functional groups can be achieved. Thus, the 2,970 and the 2,870 cm^-1^ components display zero asynchronous correlation, which implies that they originate from the same functional group. This result is consistent with the NCA, as the two normal modes display a substantial contribution from the alkyl substituent in the alpha position with respect to the nitrogen (C_18_, internal coordinates s20 and s24, respectively). The negative asynchronous correlation of both peaks with the rest of the features in the range indicates that the concentration of this group falls at a slower rate than that of the other alkyl substituents. This implies, in turn, that C_18_ is not the preferred site of initiation for the degradation process.

**FIGURE 4:**
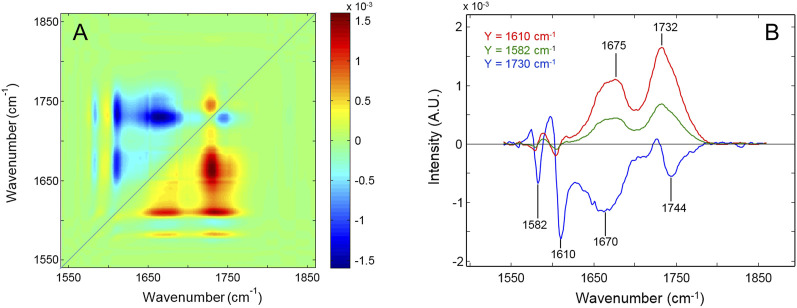
**(A)** Asynchronous spectrum in the 1,550–1,850 cm^-1^ range of the network subjected to thermo-oxidation at 180°. **(B)** Y-isofrequency plots at 1,610 cm^-1^ (red trace), 1,582 cm^-1^ (green trace), and 1,730 cm^-1^ (blue trace).

In the 1,550–1850 cm^-1^ range (Figure 4), the two sharp peaks at 1,608 and 1,582 cm^-1^ display zero asynchronous intensity according to the NCA assignments to in-plane ring modes. The carbonyl components at 1,675–1732 cm^-1^ are positively correlated with both the aromatic modes (see *Y*-isofrequency plots in Figure 4B). Considering that the synchronous spectrum is negative at the corresponding coordinates, this result indicates that aromatic group depletion starts earlier and proceeds faster than oxygen fixation on the network chains. Furthermore, the intensity is positive at (1730, 1,677), implying a faster rate of formation of the ester carbonyls compared to amides. Finally, a well-resolved correlation pattern is observed at [1745, 1730 (−)], which reveals the two-component structure of the ester band and demonstrates that the high-frequency shoulder evolves independently (more slowly) than the main component.

### 3.3 Thermal degradation kinetics

The spectra collected *in situ,* by *time-resolved* sampling, have been employed to characterize the kinetics of the degradation process. IR quantitative analysis requires the identification of isolated peaks or bands and a consistent, preferentially linear baseline. Among the numerous spectral features, those relevant to the kinetic analysis were chosen accordingly. Different methods were employed depending on the characteristics of the frequency range. Thus, the concentrations of aromatic rings and CH_2_ groups next to the ether oxygen were evaluated, respectively, from the height of the peaks at 830 and 1,510 cm^-1^. In the 3,000–2,800 cm^-1^ range, owing to the lack of resolution, integration over the entire band area was performed, which is representative of the overall concentration of alkyl groups and, therefore, monitors the degradation of the whole chain forming the 3D network. In the carbonyl region, the analysis was made by considering the difference spectra $A_{diff}\left(\nu \right)=A_{t}\left(\nu \right)-K\cdot A_{0}\left(\nu \right)$ , where *A* is the absorbance and the subscripts *diff*, *t*, and *0* refer, respectively, to the difference spectrum and to those collected at times *t* and *0*. The subtraction factor *K* was considered equal to unity for the whole data set, assuming a negligible variation of the sample thickness during the experiment.

The relative conversion, α , of a functional group is defined as (Khawam and Flanagan, 2006):

$$\alpha =\frac{C_{0}-C_{t}}{C_{0}-C_{\infty }}$$

or in terms of absorbance:

$$\alpha =\frac{A_{0}-A_{t}}{A_{0}-A_{\infty }}$$

where *C* is the molar concentration and the subscript ∞ refers to the time corresponding to final conversion.

Solid-state kinetics is usually approached by using reaction models suitably developed for describing specific processes. This is because of the heterogeneous character of the investigated phenomenon and the difficulty in obtaining information about the individual reaction steps (Khawam and Flanagan, 2006; Galwey and Brown, 1999; Jacobs and Tompkins, 1955).

The rate of a solid-state reaction can be generally described as

$$\frac{d\alpha }{dt}=P\cdot e^{-\frac{E_{a}}{RT}}\cdot f\left(\alpha \right),$$

where *P* is a pre-exponential (frequency) factor, *E*_*a*_ is the activation energy, *R* is the gas constant, *T* is the absolute temperature, and *f(α)* is the kinetic model. Kinetic parameters (*P*. *E*_*a*_, model) can be obtained from isothermal data by applying the above rate law. The photo- and thermo-oxidative processes in polymeric substrates have been successfully described by order-based models in which, analogous to homogeneous kinetics, the reaction rate is proportional to the residual reactant concentration (i.e., the conversion) raised to a certain power (integer or fractional):

$$\frac{d\alpha }{dt}=k{\left(1-\alpha \right)}^{n},$$

where *k* is an Arrhenius-type kinetic constant and *n* is the reaction order. If *n* = 0, separating the variables and integrating gives *α = kt*, while for first-order kinetics, $\alpha =1-e^{-kt}$ . Integral expressions for reaction orders 2 and 3 are available in Khawam and Flanagan (2006). Regressing the model against the experimental data provides the values of the limiting conversion (i.e., $A_{\infty }$ ). Looking at the process from the side of the products (the carbonyls), the same kinetic model identified for the depletion of the reactants is assumed, with a concentration parameter in place of a conversion (*C*_*t*_ or, equivalently, $A_{t}$ instead of *α*). Occasionally, it is observed that the kinetic profile of a specific functional group displays a more complex shape than those predicted by order-based models, suggesting that multiple independent mechanisms are operating concurrently. In these cases, the simulation of the kinetic behavior can be attempted by assuming full conversion of the reacting species by the end of the process and a sum of first-order exponential terms:

$$\alpha =\sum_{i}\alpha_{i,f}\left(1-e^{-k_{i}t}\right),$$ (1)

where each term of the summation accounts for a specific reaction pattern and $\alpha_{i,f}$ represents the limiting conversion of the *i* ^th^ mechanism.

**FIGURE 5:**
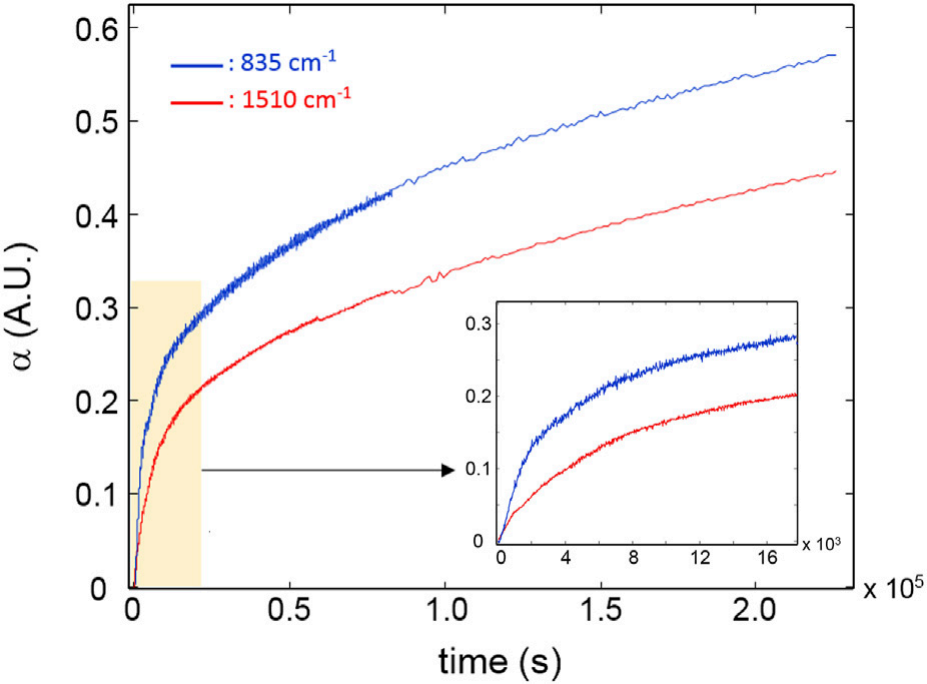
Kinetic profiles for the peaks at 835 cm^-1^ (blue curve) and 1,510 cm^-1^ (red curve). The inset highlights the early stages of the degradation process.

**FIGURE 6:**
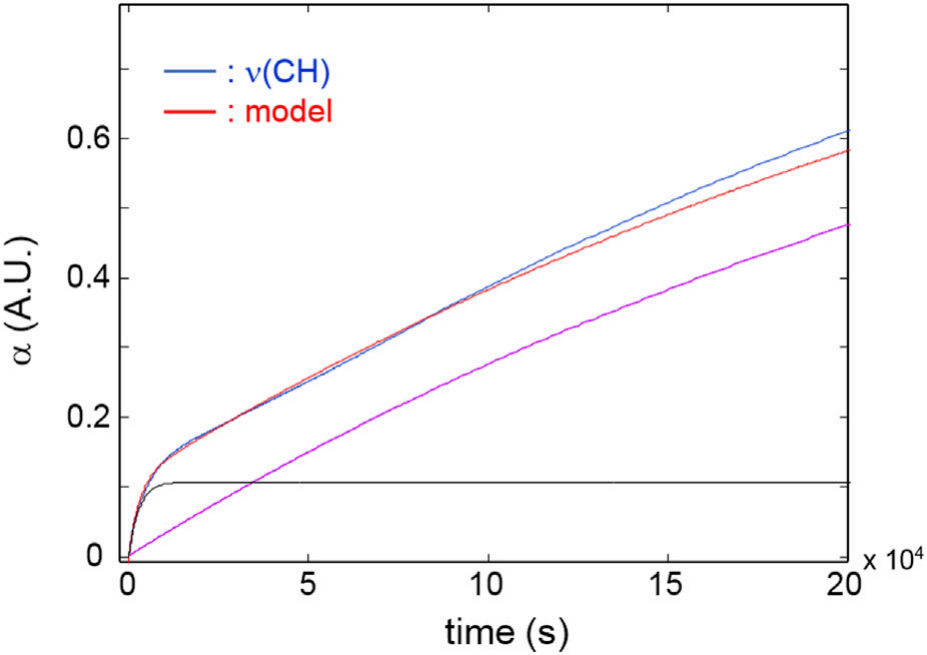
Red curve: kinetic profiles for the band area in the 3,000–2,700 cm^-1^ range. Blue curve: model simulation. Black and purple curves: simulated profiles of the exponential components.

Figure 5 reports the kinetic profiles for the aromatic rings (850 cm^-1^) and the CH_2_ next to the ether oxygen (C_11_, 1,510 cm^-1^). The single-order model is unable to simulate the two curves due to the pronounced increasing trend following the initial fast step (Figure 5). However, an excellent fit is obtained by a two component first-order model (Equation 1, *i* = 2) which provides correlation coefficients exceeding 0.995. This result suggests the occurrence of two independent reaction pathways that contribute to the depletion of the above functional groups, characterized by a largely different reaction rate (the first is more than 30 times faster than the second). A further relevant observation is that in the early stages of the process, the rate of consumption of the aromatics is considerably higher than that of the C_11_ methylenes (see inset, Figure 5), in agreement with the results of the 2D-COS analysis. Figure 6 reports the kinetic behaviour of the alkyl groups as a whole (band in the ν(CH) range) along with the simulation of the first-order, two-component model and the curves relative to the single exponentials. In this case, the fit is satisfactory (*R* ^*2*^ = 0.996) at least up to 1.2 × 10^5^ s (33 h); thereafter, the model simulation gradually departs from the experimental curve, likely due to a further complication of the degradation mechanism which possibly involves diffusion control. The analysis based on the overall concentration of alkyl groups cannot provide detailed mechanistic information but affords a robust parameter to monitor the degradation kinetics.

**FIGURE 7:**
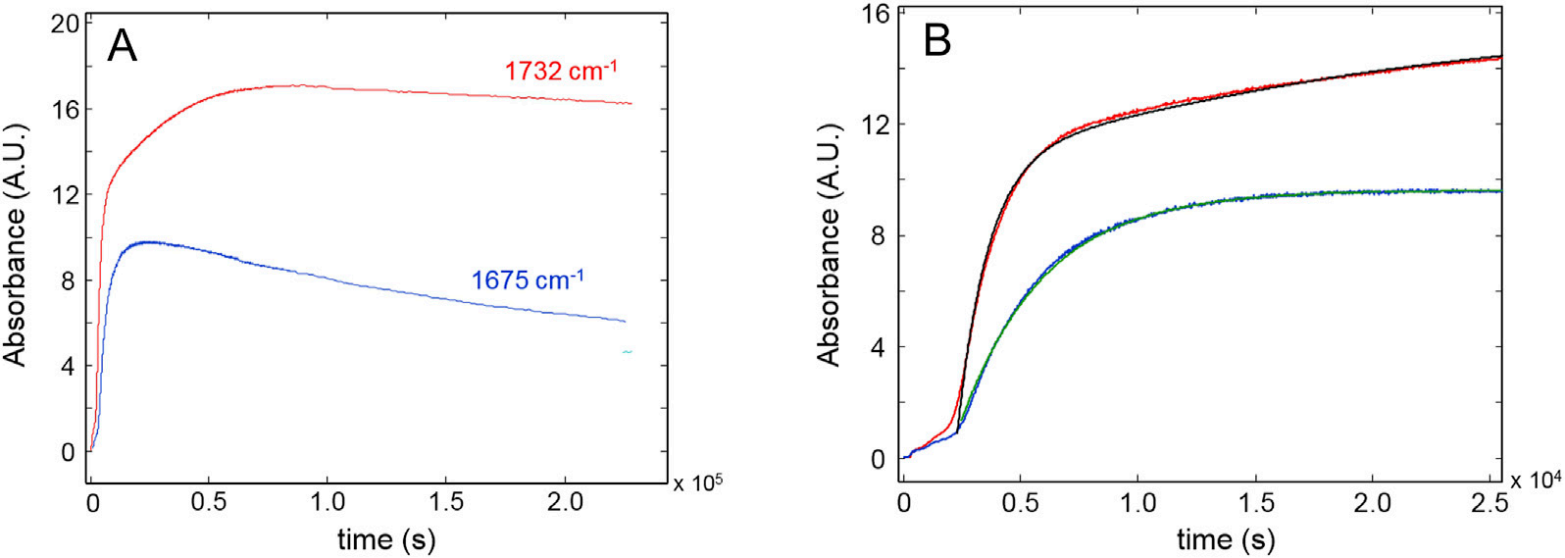
**(A)**: Kinetic profiles for the peaks at 1732 cm^-1^ (red curve) and 1,675 cm^-1^ (blue curve). **(B)** The early stages of the process. Black and green curves represent the model simulations.

Figure 7A depicts the evolution of the carbonyl bands at 1732 and 1,675 cm^-1^. Oxidative reactions producing ester and amide groups (1732 and 1,675 cm^-1^, respectively) are active in the initial 20,000 s (∼5h 30’) and proceed at a slower rate in the next 6.0 × 10^4^ s with the generation of ester groups only. Thence, the fixation of oxygen onto the network’s chains is found to end. At longer times, the concentration of carbonyls, especially those of amidic nature, is found to decrease, likely because of weight-loss steps involving these species.

Figure 7B highlights the initial period of the thermo-oxidative process, comparing the experimental data with the simulations of the kinetic models. The occurrence of an induction period lasting 3,000 s is detected for both carbonyl groups, which is not observed in the case of the conversion curves. Neglecting the initial induction period, the behavior of the amide curve can be well described by a single first-order model, at least up to the pseudo-plateau, before the decreasing trend. This suggests the prevalence of a single mechanism for the production of these groups. Conversely, the concentration of ester carbonyls is simulated by two exponential components with largely different kinetic constants (the first is 18 times larger than the second). This observation suggests that esters and amides originate from independent reaction mechanisms. Table 2 summarizes the results of the kinetic analysis in terms of rate constants and correlation coefficients.

**TABLE 2:** Kinetic parameters and correlation coefficients for the different functional groups.

Functional group	$k_{1}$ (s^-1^)	$k_{2}$ (s^-1^)	$R^{2}$
(O)CH_2_	1.8 × 10^−4^	0.8 × 10^−5^	0.999
Aromatic	3.3 × 10^−4^	1.1 × 10^−5^	0.996
CH	3.5 × 10^−4^	0.3 × 10^−5^	0.996
C=O ester	7.8 × 10^−4^	4.3 × 10^−5^	0.995
C=O amide	2.7 × 10^−4^	-	0.998

**FIGURE 8:**
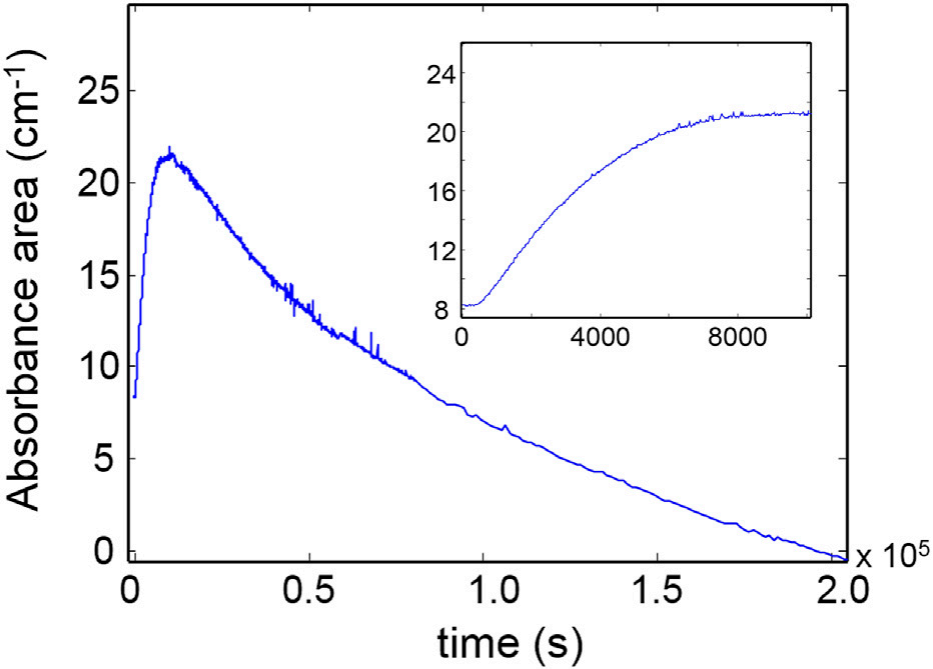
Kinetic profile for the ν(OH) band. The early stages of the degradation process are represented in the inset.

It is noted that a direct comparison between the *k* values of reagents and products is not feasible since the reaction rate for products contains additional unknown terms with respect to the rate of depletion of the reagents. However, assuming similar values for the molar absorptivity for the ν(CO) vibration of ester and amides, their rate of production can be compared. It was found that the concentration of ester groups in the network grows at a significantly higher rate than the amides. This result is in accordance with the 2D-COS analysis. Finally, Figure 8 displays the absorbance of the ν(OH) band as a function of time. The kinetic behavior is complex and cannot be described by the models adopted so far. In the early stages of the process, when the oxidative processes prevail, the concentration of hydroxyl groups increases, reaching a pseudo-plateau at around 10,000 s (see inset, Figure 8); thereafter, a second, slower mechanism induces a gradual depletion of OH groups until full loss.

### 3.4 Mechanism

In this section, we propose some of the most likely mechanisms for the thermo-oxidative degradation of the investigated network based on earlier literature studies (Musto et al., 2001; Rose et al., 1993; Musto et al., 2012; Musto, 2003; Rivaton et al., 1997a; Rivaton et al., 1997b) and the present experimental evidence, which can be summarized as follows.

i) The methylene and methyl groups are both involved in the chain-scission process through concurrent and competitive reaction pathways. These groups likely represent the main sites of initiation. ii) The aromatic ring of DGEBA is involved in a fast process which causes the loss of aromaticity; in fact, a pathway limited to modifying the ring substitution would produce a frequency shift of the associated peaks rather than the observed intensity decrease. iii) Oxygen is fixed on the network chains through the formation of amide and ester groups, the latter species being produced at a faster rate and in a larger amount. 2D-COS analysis unambiguously identified two distinct ester groups evolving at different rates.

The thermo-oxidation of polymers initiates with the formation of radical species (frequently from impurities) that are able to abstract hydrogen from a backbone chain (PH) (Rivaton et al., 1997a), forming a macroradical (P·). The latter reacts with oxygen to form hydroperoxides, which initiate the so-called basic autoxidation scheme (BAS) (Kelen, 1983).

**SCHEME 2:**
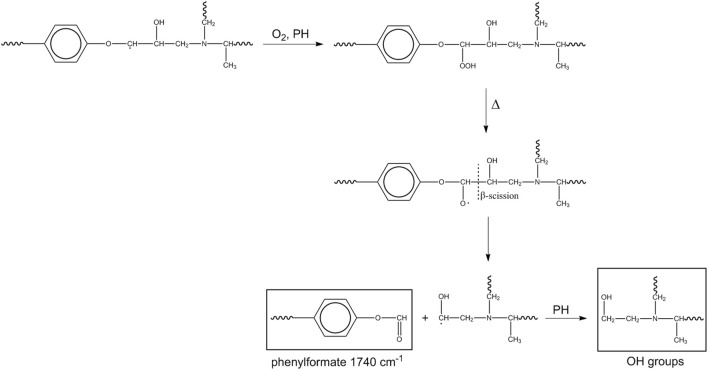
Mechanism 1.

A first pathway (Scheme 2) starts at the methylene of the alkyl-aryl ether.

**SCHEME 3:**
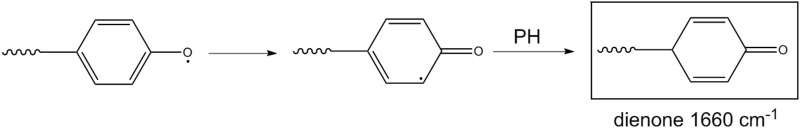
Mechanism 2.

This mechanism accounts for the production of hydroxyls in the early stages of the process and for the high-frequency shoulder of the carbonyl band (phenylformate). If the β-scission occurs on the opposite site (toward the ether oxygen), the macroradical thus formed evolves by hydrogen abstraction from PH, with the formation of a cyclic dienone and loss of the aromatic character of the aryl group (Scheme 3).

This route accounts for the observed depletion of aromatic groups and suggests that the signals in the 1,700–1,630 cm^-1^ range may not originate solely from amides but also from a conjugated ketonic structure.

Another mechanism initiates at the methylene next to the nitrogen.

**SCHEME 4:**
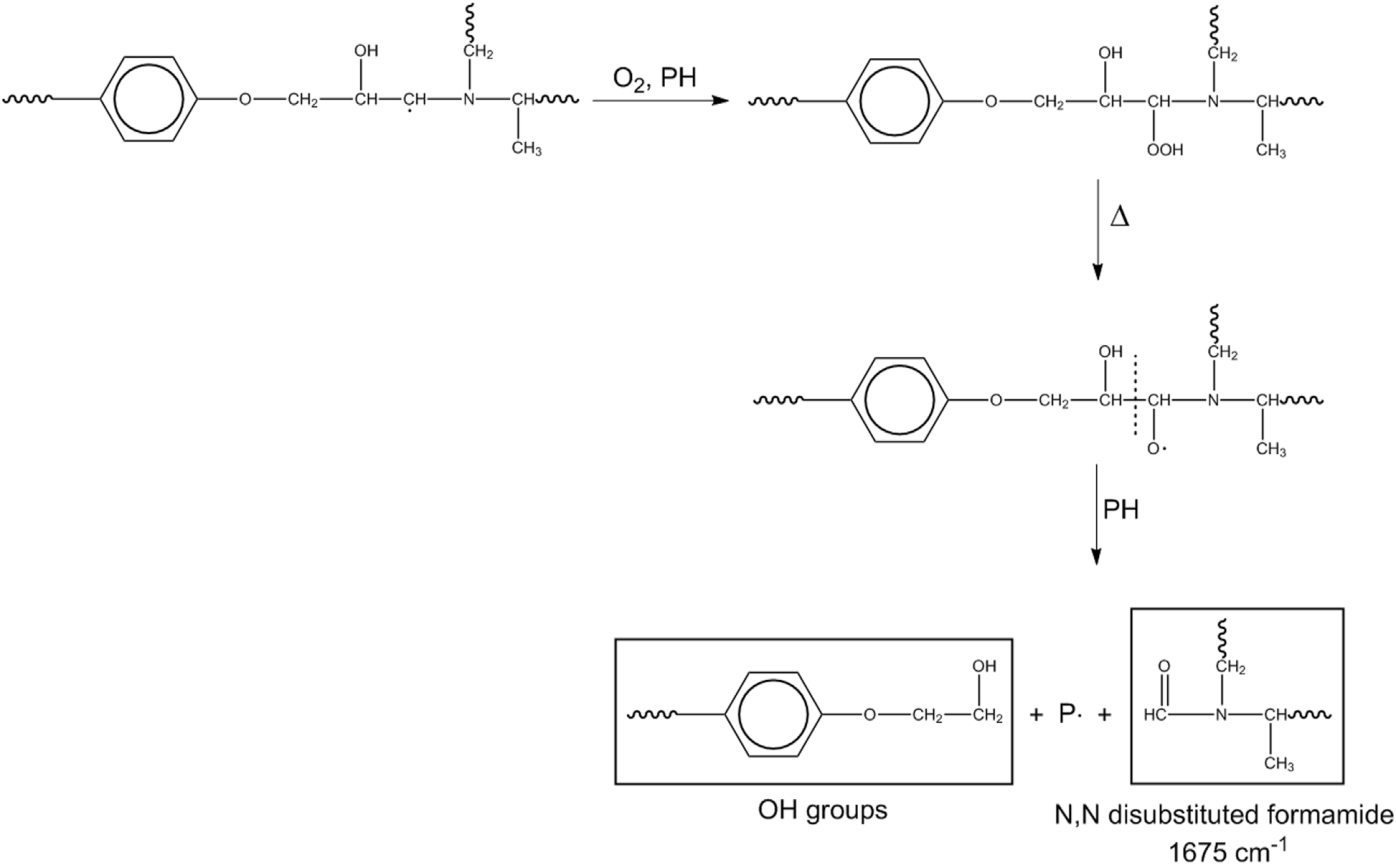
Mechanism 3.

This pathway produces the amide linkages (N,N-disubstituted formamide) and further hydroxyls (Scheme 4).

**SCHEME 5:**
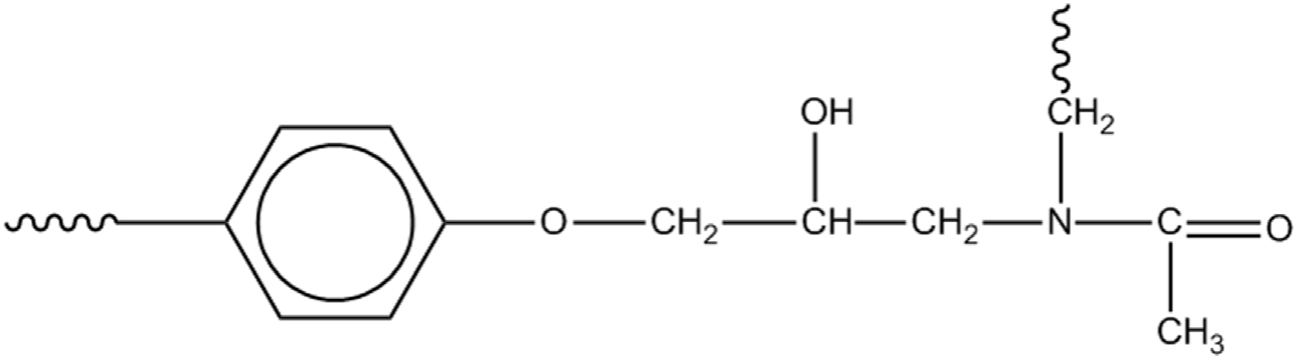
N,N-disubstituted acetamide.

A further amidic species can be formed by initiation at the methylene next to the nitrogen, leading to Scheme 5.

**SCHEME 6:**
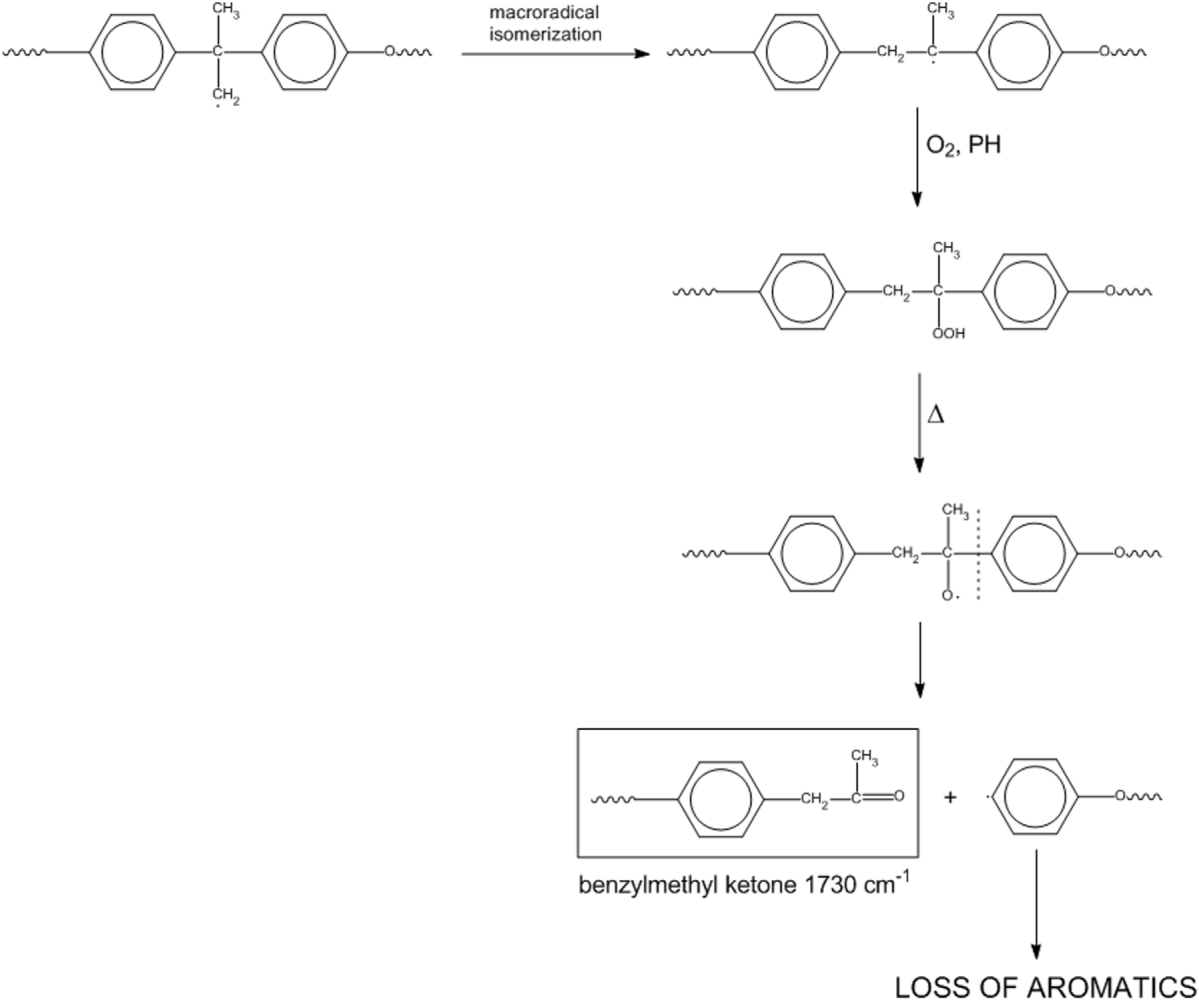
Mechanism 4.

If initiation starts at the isopropylidene site, we may have Scheme 6.

This mechanism represents a second route for the loss of aromatics and accounts for the peak observed at 1732 cm^-1^ (benzylmethyl-ketone).

In the light of the kinetic analysis and of the 2D-COS results, Mechanisms 1 and 4 seem to be the preferred paths, since the consumption of the methylenes of the alkylaryl ether moiety is faster than the consumption of alkyl groups bound to the nitrogen atom and because the keto groups develop faster than the amides. In particular, Mechanism 4 is favored over Mechanism 1, as demonstrated by 2D-COS in the carbonyl range. The higher rate of depletion of aromatic groups compared to different alkyl units may be ascribed to multiple reaction pathways, leading to loss of aromaticity (Mechanisms 1 and 4).

## 4 Conclusion

We performed *in situ*, FTIR measurements in the *time-resolved* mode under controlled conditions of temperature and environment to elucidate the kinetics and mechanisms of the thermo-oxidative degradation of a flexible epoxy network.

The main findings of the present contribution can be summarized thus.

• The NC analysis routed on the DFT method allowed a reliable interpretation of the complex infrared spectrum of the network, improving the level of structural detail that can be accessed by this spectroscopic technique.
• The relative stability of the various functional groups was ranked. In agreement with previous literature, it was found that initiation occurs at the aliphatic moieties and at the isopropylidene unit. Aryl groups are also involved with loss of the aromatic character. The oxidation of the network chains produces amide and ketone groups, with the latter developing at a higher rate.
• The kinetic behavior of the system was suitably simulated by a first-order, biexponential model, which afforded the evaluation of the kinetic constants for the main functional groups involved in the process.
• On the basis of the spectroscopic analysis, the most likely thermo-oxidative mechanisms were proposed and discussed. The 2D-COS results coupled with the kinetic analysis allowed us to identify those that occur preferentially.

## Data Availability

The raw data supporting the conclusions of this article will be made available by the authors, without undue reservation.
