# Supplementary material for: In-situ FTIR spectroscopy of epoxy resin degradation: kinetics and mechanisms
Source: Front Chem. 2024 Oct 30;12:1476965. doi: 10.3389/fchem.2024.1476965 (PMC11557421; doi:10.3389/fchem.2024.1476965)
Supplement: Supplementary file 1 [file DataSheet1.PDF]

In-Situ FTIR Spectroscopy of Epoxy Resin Degradation: Kinetics and Mechanisms

Marianna Pannico[1], Giuseppe Mensitieri[2], Pellegrino Musto[1, *]

[1]: National Research Council of Italy, Institute of Polymers, Composites and Biomaterials, via Campi Flegrei, 34, 80078, Pozzuoli, Italy

[2]: Department of Chemical, Materials and Production Engineering, University of Naples Federico II, P.le Tecchio 80, 80125 Naples,Italy.

Supplementary Material

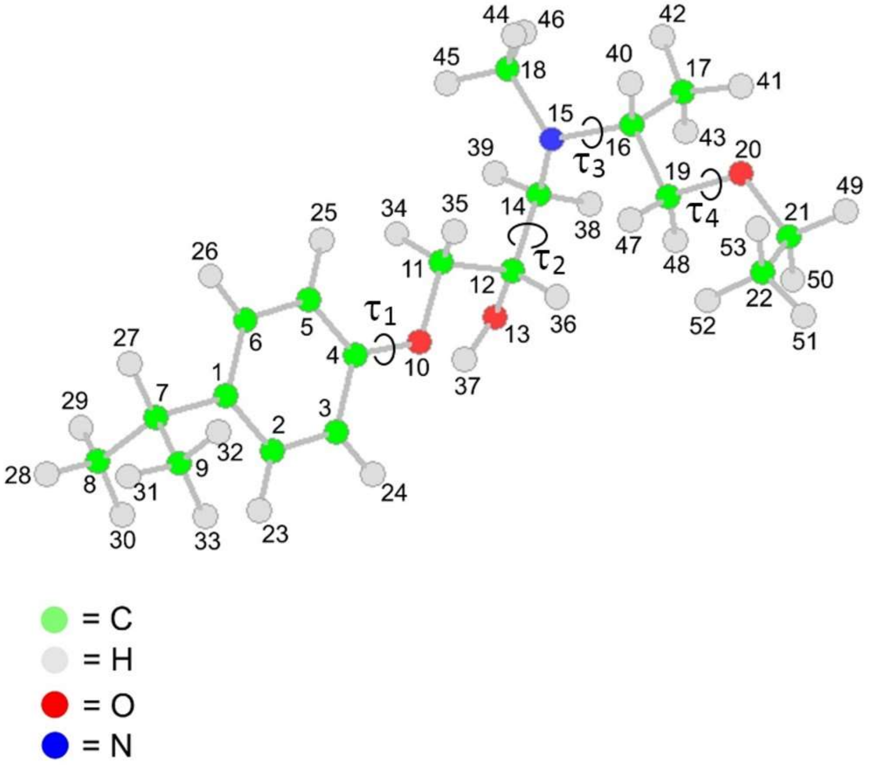

Figure S1. *Molecular structure of the model compound relaxed at the B3LYP/3-21G level of theory, with atom numbering and the definition of the four conformational degrees of freedom.*

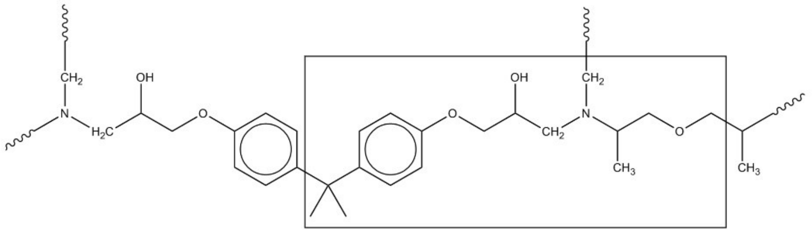

Figure S2. *Network structure and the molecular fragment chosen as model compound.*

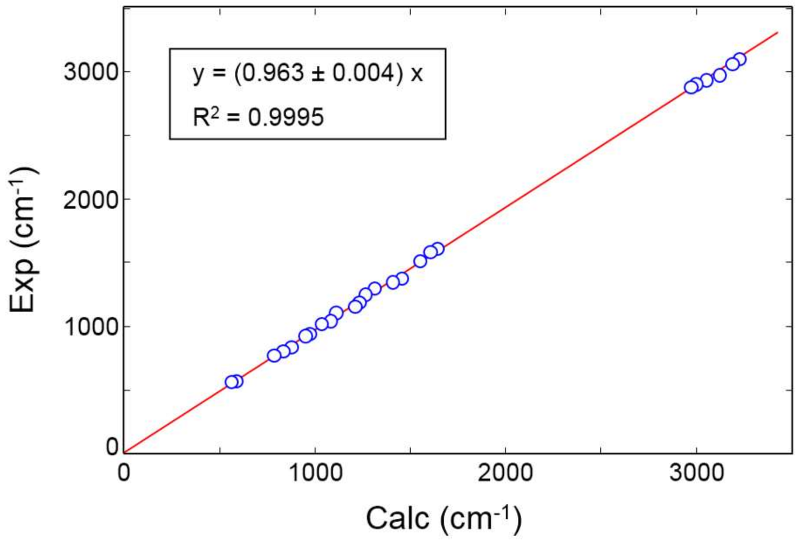

Figure S3. *Experimental vs calculated frequencies for the molecular model of the epoxy network.*

Table S1. Non-redundant internal coordinate definition

Name	Fac	Type	atom	n.	Sym	value	PED
s1	1.00	STRE	13	37	OH	0.997889	f3453 100
s2	1.00	STRE	2	23	CH	1.084375	f3194 91
s3	1.00	STRE	3	24	CH	1.082398	f3225 90
s4	1.00	STRE	5	25	CH	1.081533	f3232 95
s5	1.00	STRE	6	26	CH	1.085188	f3186 96
s6	1.00	STRE	7	27	CH	1.097937	f3048 82
s7	1.00	STRE	8	29	CH	1.094817	f3120 83
	-1.00		9	32	CH	1.094823	
	1.00		9	33	CH	1.095807	
s8	-1.00	STRE	8	28	CH	1.096359	f3113 78
	1.00		8	29	CH	1.094817	
	1.00		8	30	CH	1.095789	
	-1.00		9	31	CH	1.096369	
s9	-1.00	STRE	8	28	CH	1.096359	f3044 84
	-1.00		8	29	CH	1.094817	
	-1.00		8	30	CH	1.095789	
	1.00		9	31	CH	1.096369	
	1.00		9	32	CH	1.094823	
s10	-1.00	STRE	8	28	CH	1.096359	f3105 83
	1.00		9	31	CH	1.096369	
	-1.00		9	33	CH	1.095807	
s11	1.00	STRE	8	29	CH	1.094817	f3123 85
	-1.00		8	30	CH	1.095789	
s12	-1.00	STRE	11	34	CH	1.096808	f3049 60
	-1.00		11	35	CH	1.091745	
	1.00		8	30	CH	1.095789	
s13	1.00	STRE	16	40	CH	1.096376	f3075 80
	1.00		18	45	CH	1.094590	
	1.00		19	47	CH	1.096899	

s14	1.00	STRE	11	35	CH	1.091745	f3133 85	f3049 10
s15	1.00	STRE	12	36	CH	1.098908	f3027 81	
s16	1.00	STRE	9	33	CH	1.095807	f3049 69	
	1.00		8	30	CH	1.095789		
	1.00		8	29	CH	1.094817		
	1.00		11	34	CH	1.096808		
	1.00		11	35	CH	1.091745		
s17	-1.00	STRE	19	47	CH	1.096899	f3060 12	f3052 59
	1.00		14	38	CH	1.096645		
	1.00		16	40	CH	1.096376		
	1.00		14	39	CH	1.095773		
s18	1.00	STRE	14	38	CH	1.096645	f3097 84	
	-1.00		14	39	CH	1.095773		
s19	1.00	STRE	17	41	CH	1.092298	f3153 91	
s20	1.00	STRE	14	39	CH	1.095773	f3121 77	
	1.00		16	40	CH	1.096376		
	1.00		17	42	CH	1.094607		
	-1.00		17	43	CH	1.095848		
	-1.00		18	44	CH	1.095196		
	1.00		18	45	CH	1.094590		
s21	1.00	STRE	17	41	CH	1.092298	f3062 74	
	1.00		17	42	CH	1.094607		
	1.00		17	43	CH	1.095848		
	1.00		21	49	CH	1.093797		
	1.00		21	50	CH	1.100914		
s22	-1.00	STRE	16	40	CH	1.096376	f3070 85	
	1.00		17	41	CH	1.092298		
	1.00		17	42	CH	1.094607		
	1.00		18	44	CH	1.095196		
	1.00		18	45	CH	1.094590		
	-1.00		19	47	CH	1.096899		
	1.00		21	49	CH	1.093797		
	1.00		21	50	CH	1.100914		
s23	1.00	STRE	18	44	CH	1.095196	f3121 11	f3114 83
	-1.00		18	45	CH	1.094590		
s24	1.00	STRE	18	46	CH	1.103543	f2969 94	
s25	1.00	STRE	14	38	CH	1.096645	f3062 10	f3047 74
	1.00		14	39	CH	1.095773		
	1.00		19	47	CH	1.096899		
s26	1.00	STRE	19	47	CH	1.096899	f3004 10	f2994 75
	1.00		19	48	CH	1.101229		
	-1.00		21	50	CH	1.100914		
s27	1.00	STRE	21	49	CH	1.093797	f3095 79	
s28	1.00	STRE	21	50	CH	1.100914	f3004 83	f2994 11
s29	1.00	STRE	22	51	CH	1.095543	f3123 84	
s30	1.00	STRE	22	52	CH	1.094657	f3136 86	f3095 11
	-1.00		22	53	CH	1.094051		
s31	1.00	STRE	22	51	CH	1.095543	f3060 78	f3052 11
	1.00		22	52	CH	1.094657		
	1.00		22	53	CH	1.094051		
s32	-1.00	STRE	4	5	CC	1.394954	f1606 50	
	1.00		1	6	CC	1.397258		
s33	-1.00	STRE	2	3	CC	1.388418	f1646 52	f1233 13
	-1.00		10	4	OC	1.390155		
	1.00		1	6	CC	1.397258		
	1.00		4	5	CC	1.394954		
s34	1.00	STRE	3	4	CC	1.402014	f839 38	
	1.00		2	3	CC	1.388418		
	1.00		10	4	OC	1.390155		
	1.00		10	11	OC	1.472131		
	1.00		1	6	CC	1.397258		
s35	1.00	STRE	13	12	OC	1.454677	f1108 27	f947 28
s36	1.00	STRE	10	4	OC	1.390155	f1265 46	

s37	1.00	STRE	5	6		CC	1.398207	f1469 11 f1248 10 f1158 27
	1.00		1	6		CC	1.397258	
s38	1.00	STRE	7	1		CC	1.526515	f1248 25 f725 18
s39	1.00	STRE	16	17		CC	1.544680	f867 42 f865 10
	1.00		20	21		OC	1.461725	
	1.00		21	22		CC	1.535989	
	1.00		20	19		OC	1.458806	
	1.00		16	19		CC	1.537191	
s40	1.00	STRE	19	16		CC	1.537191	f1178 10 f1092 26
	-1.00		12	11		CC	1.525208	
	-1.00		17	16		CC	1.544680	
	1.00		14	12		CC	1.531679	
s41	1.00	STRE	15	14		NC	1.485205	f1008 32
	-1.00		15	16		NC	1.487925	
s42	-1.00	STRE	12	14		CC	1.531679	f1108 16 f1082 23 f1037 14
	1.00		20	21		OC	1.461725	
	1.00		11	12		CC	1.525208	
s43	1.00	STRE	21	22		CC	1.535989	f973 65 f959 11
	-1.00		20	19		OC	1.458806	
s44	1.00	STRE	15	18		NC	1.480220	f1082 13 f1037 20
s45	-1.00	STRE	15	16		NC	1.487925	f787 62
	-1.00		15	14		NC	1.485205	
	-1.00		16	17		CC	1.544680	
	-1.00		15	18		NC	1.480220	
	1.00		20	21		OC	1.461725	
	1.00		20	19		OC	1.458806	
s46	1.00	STRE	16	17		CC	1.544680	f880 50
	1.00		16	19		CC	1.537191	
	1.00		11	12		CC	1.525208	
	-1.00		15	14		NC	1.485205	
	-1.00		21	22		CC	1.535989	
	-1.00		20	21		OC	1.461725	
s47	-1.00	STRE	9	7		CC	1.548886	f1132 33 f982 29 f959 23
	1.00		8	7		CC	1.548835	
s48	1.00	STRE	8	7		CC	1.548835	f894 59
	1.00		9	7		CC	1.548886	
s49	1.00	STRE	20	19		OC	1.458806	f1141 13 f1068 51
	1.00		16	19		CC	1.537191	
	-1.00		20	21		OC	1.461725	
	-1.00		12	14		CC	1.531679	
s50	1.00	STRE	10	11		OC	1.472131	f1018 56 f1008 11
s51	1.00	STRE	1	6		CC	1.397258	f1358 10 f1313 57
	1.00		4	5		CC	1.394954	
	1.00		2	3		CC	1.388418	
	-1.00		3	4		CC	1.402014	
	-1.00		10	4		OC	1.390155	
s52	1.00	STRE	21	22		CC	1.535989	f867 11 f865 21
	1.00		20	19		OC	1.458806	
	1.00		12	14		CC	1.531679	
	1.00		11	12		CC	1.525208	
	1.00		20	21		OC	1.461725	
	1.00		16	19		CC	1.537191	
	1.00		16	17		CC	1.544680	
	1.00		15	18		NC	1.480220	
k53	1.00	BEND	2	3	4	CCC	120.38	f1051 61
	1.00		4	5	6	CCC	119.83	
s54	1.00	BEND	37	13	12	HOC	104.28	f1459 31 f1334 11 f1299 15 f1037 10
s55	-1.00	BEND	34	11	35	HCH	110.52	f1554 53
	-1.00		25	5	6	HCC	119.49	
	1.00		24	3	4	HCC	118.21	
s56	-1.00	BEND	26	6	1	HCC	119.32	f1469 46 f1158 29
	1.00		24	3	4	HCC	118.21	
	-1.00		23	2	3	HCC	119.01	

s57	1.00	BEND	25	5	6	HCC	119.49	f1646 16	f1265 11	f1233 40	
	-1.00		26	6	1	HCC	119.32				
	-1.00		24	3	4	HCC	118.21				
	1.00		23	2	3	HCC	119.01				
s58	1.00	BEND	24	3	4	HCC	118.21	f1408 64	f1248 10		
	-1.00		27	7	9	HCC	108.09				
s59	1.00	BEND	23	2	3	HCC	119.01	f1606 16	f1358 60		
	1.00		26	6	1	HCC	119.32				
	1.00		27	7	9	HCC	108.09				
	1.00		24	3	4	HCC	118.21				
	1.00		25	5	6	HCC	119.49				
s60	-1.00	BEND	32	9	33	HCH	108.32	f1553 70	f982 10		
	1.00		31	9	32	HCH	108.76				
s61	1.00	BEND	29	8	30	HCH	108.32	f1574 50			
	1.00		32	9	33	HCH	108.32				
	-1.00		28	8	29	HCH	108.77				
	-1.00		31	9	32	HCH	108.76				
	-1.00		25	5	6	HCC	119.49				
	1.00		23	2	3	HCC	119.01				
	-1.00		26	6	1	HCC	119.32				
	1.00		24	3	4	HCC	118.21				
s62	1.00	BEND	28	8	30	HCH	108.25	f1448 96			
	1.00		29	8	30	HCH	108.32				
	-1.00		31	9	32	HCH	108.76				
	-1.00		31	9	33	HCH	108.25				
s63	1.00	BEND	28	8	29	HCH	108.77	f1465 89			
	1.00		28	8	30	HCH	108.25				
	1.00		29	8	30	HCH	108.32				
	1.00		31	9	32	HCH	108.76				
	1.00		31	9	33	HCH	108.25				
	1.00		32	9	33	HCH	108.32				
s64	1.00	BEND	28	8	29	HCH	108.77	f1567 65	f1203 14		
	1.00		31	9	32	HCH	108.76				
	-1.00		28	8	30	HCH	108.25				
	-1.00		31	9	33	HCH	108.25				
s65	-1.00	BEND	31	9	33	HCH	108.25	f1555 71	f959 14		
	1.00		28	8	30	HCH	108.25				
	1.00		31	9	32	HCH	108.76				
	-1.00		28	8	29	HCH	108.77				
s66	1.00	BEND	47	19	20	HCO	112.31	f1379 12	f1278 13	f1229 12	f1211 10
s67	1.00	BEND	34	11	35	HCH	110.52	f1568 51			
	1.00		28	8	30	HCH	108.25				
	1.00		31	9	33	HCH	108.25				
	-1.00		25	5	6	HCC	119.49				
	1.00		24	3	4	HCC	118.21				
s68	-1.00	BEND	47	19	20	HCO	112.31	f1257 37	f1229 12		
	-1.00		34	11	10	HCO	109.82				
	1.00		36	12	13	HCO	111.80				
s69	1.00	BEND	38	14	15	HCN	112.66	f1383 10	f1334 36		
s70	1.00	BEND	42	17	43	HCH	108.69	f1552 62			
	-1.00		44	18	45	HCH	108.07				
	-1.00		41	17	42	HCH	109.54				
	-1.00		47	19	48	HCH	108.80				
s71	1.00	BEND	49	21	22	HCC	110.15	f1342 58			
s72	1.00	BEND	38	14	39	HCH	106.80	f1549 68			
	-1.00		49	21	50	HCH	108.50				
s73	1.00	BEND	44	18	46	HCH	108.27	f1584 68			
	-1.00		45	18	46	HCH	108.15				
	1.00		41	17	43	HCH	108.45				
	1.00		42	17	43	HCH	108.69				
	1.00		47	19	48	HCH	108.80				
s74	1.00	BEND	42	17	41	HCH	109.54	f1443 46	f1428 20		
	1.00		41	17	43	HCH	108.45				

	1.00		43	17	42	HCH	108.69	
s75	-1.00	BEND	41	17	43	HCH	108.45	f1574 48 f1574 11
	1.00		41	17	42	HCH	109.54	
	1.00		34	11	35	HCH	110.52	
	1.00		44	18	46	HCH	108.27	
s76	1.00	BEND	44	18	45	HCH	108.07	f1560 61
	-1.00		45	18	46	HCH	108.15	
	-1.00		41	17	43	HCH	108.45	
	-1.00		41	17	42	HCH	109.54	
s77	1.00	BEND	44	18	45	HCH	108.07	f1499 75
	1.00		45	18	46	HCH	108.15	
s78	1.00	BEND	36	12	13	HCO	111.80	f1459 10 f1420 15 f1379 10 f1299 12
	1.00		47	19	20	HCO	112.31	
s79	1.00	BEND	47	19	48	HCH	108.80	f1581 39 f1443 10
	-1.00		41	17	42	HCH	109.54	
	-1.00		41	17	43	HCH	108.45	
s80	1.00	BEND	40	16	19	HCC	105.66	f1411 16 f1404 15 f1379 20 f1372 11
s81	1.00	BEND	49	21	50	HCH	108.50	f1549 69
	1.00		38	14	39	HCH	106.80	
s82	-1.00	BEND	52	22	53	HCH	108.60	f1581 12 f1568 44
	1.00		51	22	52	HCH	108.24	
s83	1.00	BEND	51	22	52	HCH	108.24	f1556 69
	-1.00		51	22	53	HCH	108.94	
s84	1.00	BEND	50	21	49	HCH	108.50	f1456 87
	1.00		51	22	53	HCH	108.94	
	1.00		52	22	51	HCH	108.24	
	1.00		53	22	52	HCH	108.60	
s85	1.00	BEND	3	4	5	CCC	119.23	f1051 16 f725 22
s86	1.00	BEND	1	6	5	CCC	121.66	f676 67
	1.00		2	3	4	CCC	120.38	
s87	1.00	BEND	1	6	5	CCC	121.66	f1646 11 f1097 12 f590 21
	1.00		4	5	6	CCC	119.83	
s88	1.00	BEND	17	16	15	CCN	115.87	f377 35
	1.00		12	14	15	CCN	111.40	
	1.00		5	4	10	CCO	124.55	
s89	-1.00	BEND	8	7	9	CCC	110.58	f319 56
	1.00		6	1	7	CCC	120.95	
	-1.00		12	11	10	CCO	103.39	
s90	1.00	BEND	6	1	7	CCC	120.95	f195 47
	1.00		12	14	15	CCN	111.40	
	1.00		12	11	10	CCO	103.39	
	-1.00		14	12	13	CCO	106.12	
	-1.00		4	10	11	COC	119.26	
	1.00		16	19	20	CCO	105.52	
s91	1.00	BEND	14	12	11	CCC	111.41	f1037 11
s92	1.00	BEND	14	15	18	CNC	113.89	f205 13 f117 11
	1.00		6	1	7	CCC	120.95	
	-1.00		12	14	15	CCN	111.40	
	-1.00		19	20	21	COC	113.72	
	1.00		4	10	11	COC	119.26	
s93	1.00	BEND	14	15	16	CNC	116.26	f543 18 f398 11 f272 12 f241 11 f231 11
s94	1.00	BEND	5	4	10	CCO	124.55	f507 15 f231 11
	-1.00		17	16	15	CCN	115.87	
	-1.00		12	14	15	CCN	111.40	
s95	1.00	BEND	22	21	20	CCO	112.77	f443 10 f437 20 f301 11
s96	1.00	BEND	14	15	18	CNC	113.89	f447 39
	1.00		19	16	15	CCN	110.20	
s97	1.00	BEND	17	16	15	CCN	115.87	f350 27 f241 10
	1.00		19	16	15	CCN	110.20	
s98	1.00	BEND	6	1	7	CCC	120.95	f319 11
	-1.00		12	11	10	CCO	103.39	
s99	1.00	BEND	8	7	1	CCC	111.23	f570 10 f434 16 f248 15

s100	-1.00	BEND	11	10	4		COC	119.26	f51 14
	1.00		13	12	14		OCC	106.12	
	-1.00		21	20	19		COC	113.72	
s101	1.00	BEND	16	19	20		CCO	105.52	f562 40
	-1.00		12	14	15		CCN	111.40	
	1.00		19	20	21		COC	113.72	
	-1.00		14	12	13		CCO	106.12	
s102	1.00	BEND	14	12	13		CCO	106.12	f398 10 f123 19
	-1.00		16	19	20		CCO	105.52	
s103	1.00	BEND	14	12	13		CCO	106.12	f398 13 f272 27
	1.00		16	19	20		CCO	105.52	
s104	1.00	TORS	37	13	12	11	HOCC	44.15	f525 66 f507 10
s105	1.00	TORS	23	2	1	7	HCCC	0.02	f1006 78
	1.00		24	3	4	5	HCCC	-180.14	
s106	-1.00	TORS	23	2	1	7	HCCC	0.02	f855 97
	1.00		24	3	4	5	HCCC	-180.14	
	-1.00		25	5	6	1	HCCC	-180.15	
	-1.00		26	6	1	7	HCCC	-0.02	
s107	-1.00	TORS	23	2	1	7	HCCC	0.02	f876 64 f570 22
	1.00		26	6	1	7	HCCC	-0.02	
	1.00		24	3	4	5	HCCC	-180.14	
	1.00		25	5	6	1	HCCC	-180.15	
s108	1.00	TORS	26	6	1	7	HCCC	-0.02	f1000 62 f781 10 f443 15
	-1.00		25	5	6	1	HCCC	-180.15	
s109	1.00	TORS	27	7	1	2	HCCC	-180.07	f1370 69 f982 12
s110	1.00	TORS	28	8	7	9	HCCC	-58.19	f1567 16 f1248 10 f1203 39
	-1.00		31	9	7	8	HCCC	58.18	
	-1.00		29	8	7	9	HCCC	-178.67	
	-1.00		30	8	7	9	HCCC	61.43	
s111	1.00	TORS	29	8	7	9	HCCC	-178.67	f1553 12 f1132 15 f982 33 f237 14
	1.00		32	9	7	8	HCCC	-181.34	
s112	1.00	TORS	28	8	7	9	HCCC	-58.19	f263 82
	1.00		29	8	7	9	HCCC	-178.67	
	1.00		30	8	7	9	HCCC	61.43	
	-1.00		31	9	7	8	HCCC	58.18	
	-1.00		32	9	7	8	HCCC	-181.34	
s113	1.00	TORS	28	8	7	9	HCCC	-58.19	f1555 14 f959 29 f248 10 f237 16
	1.00		31	9	7	8	HCCC	58.18	
s114	-1.00	TORS	33	9	7	8	HCCC	-61.44	f1574 10 f1097 35
	1.00		30	8	7	9	HCCC	61.43	
	-1.00		29	8	7	9	HCCC	-178.67	
	1.00		32	9	7	8	HCCC	-181.34	
s115	1.00	TORS	30	8	7	9	HCCC	61.43	f1132 20 f959 19 f248 23
	1.00		33	9	7	8	HCCC	-61.44	
s116	1.00	TORS	34	11	10	4	HCOC	61.77	f1383 20 f1082 13
s117	1.00	TORS	35	11	10	4	HCOC	-61.46	f1383 22 f1257 13 f947 11
s118	1.00	TORS	38	14	15	16	HCNC	19.03	
s119	1.00	TORS	39	14	15	16	HCNC	-221.83	f1372 15 f1334 11
s120	1.00	TORS	40	16	15	14	HCNC	-180.78	f1411 45
s121	1.00	TORS	41	17	16	19	HCCC	52.48	f1552 11 f959 11
	-1.00		42	17	16	19	HCCC	-187.34	
s122	1.00	TORS	43	17	16	19	HCCC	-66.10	f248 67 f241 14
s123	1.00	TORS	43	17	16	19	HCCC	-66.10	f959 27
	-1.00		41	17	16	19	HCCC	52.48	
	-1.00		47	19	20	21	HCOC	-70.25	
s124	-1.00	TORS	45	18	15	14	HCNC	-53.82	f1560 17 f1162 53
	1.00		44	18	15	14	HCNC	-171.62	
s125	1.00	TORS	44	18	15	14	HCNC	-171.62	f1584 20 f1092 16
	-1.00		46	18	15	14	HCNC	67.38	
s126	1.00	TORS	46	18	15	14	HCNC	67.38	f241 11 f217 12 f205 59
	1.00		45	18	15	14	HCNC	-53.82	
	1.00		44	18	15	14	HCNC	-171.62	
s127	-1.00	TORS	41	17	16	19	HCCC	52.48	f1187 11

	1.00		43	17	16	19	HCCC	-66.10	
	1.00		47	19	20	21	HCOC	-70.25	
s128	1.00	TORS	48	19	20	21	HCOC	51.37	f1404 19 f1278 15
s129	1.00	TORS	49	21	20	19	HCOC	-166.26	f1556 10 f1187 12 f301 16
	-1.00		51	22	21	20	HCCO	-186.35	
s130	-1.00	TORS	49	21	20	19	HCOC	-166.26	f1428 19 f1404 24
	1.00		50	21	20	19	HCOC	-49.73	
s131	1.00	TORS	49	21	20	19	HCOC	-166.26	f1556 11 f833 67
	1.00		51	22	21	20	HCCO	-186.35	
	-1.00		53	22	21	20	HCCO	53.86	
s132	1.00	TORS	52	22	21	20	HCCO	-66.28	f1141 16 f1133 10
s133	1.00	TORS	53	22	21	20	HCCO	53.86	f1187 12
s134	1.00	TORS	1	6	5	4	CCCC	0.01	f1000 23 f781 21 f443 29 f437 10
s135	1.00	TORS	5	4	3	2	CCCC	-0.03	f781 21 f437 13 f434 15
s136	-1.00	TORS	3	4	10	11	CCOC	-179.04	f98 23 f91 12 f51 14
	-1.00		4	10	11	12	COCC	-180.69	
	1.00		11	12	14	15	CCCN	-55.71	
	-1.00		12	14	15	16	CCNC	-103.32	
	1.00		19	20	21	22	COCC	74.03	
	1.00		15	16	19	20	NCCO	-189.33	
s137	1.00	TORS	4	10	11	12	COCC	-180.69	f9 66
	1.00		12	14	15	16	CCNC	-103.32	
	1.00		11	12	14	15	CCCN	-55.71	
	-1.00		14	12	11	10	CCCO	-172.54	
s138	1.00	TORS	14	12	11	10	CCCO	-172.54	f153 43
	-1.00		3	4	10	11	CCOC	-179.04	
	1.00		12	14	15	16	CCNC	-103.32	
s139	1.00	TORS	19	16	15	14	CCNC	65.29	f91 14 f69 23
s140	1.00	TORS	19	20	21	22	COCC	74.03	f170 45 f123 11
	-1.00		15	16	19	20	NCCO	-189.33	
	-1.00	OUT	18	14	16	15	CCCN	41.55	
s141	-1.00	TORS	2	1	7	8	CCCC	61.80	f44 65
	1.00		11	12	14	15	CCCN	-55.71	
s142	1.00	TORS	16	19	20	21	CCOC	-188.89	f30 59
	-1.00		4	10	11	12	COCC	-180.69	
	1.00		12	14	15	16	CCNC	-103.32	
s143	1.00	TORS	2	1	7	8	CCCC	61.80	f10 62
	1.00		3	4	10	11	CCOC	-179.04	
	1.00		11	12	14	15	CCCN	-55.71	
s144	1.00	TORS	19	20	21	22	COCC	74.03	f74 54
	1.00		15	16	19	20	NCCO	-189.33	
s145	-1.00	TORS	16	19	20	21	CCOC	-188.89	f74 10 f31 39
	-1.00		4	10	11	12	COCC	-180.69	
	1.00		12	14	15	16	CCNC	-103.32	
s146	1.00	TORS	4	10	11	12	COCC	-180.69	f98 29 f91 10 f51 11 f31 10
	-1.00		11	12	14	15	CCCN	-55.71	
	1.00		3	4	10	11	CCOC	-179.04	
	1.00		3	4	5	6	CCCC	0.03	
	-1.00		19	20	21	22	COCC	74.03	
	-1.00		15	16	19	20	NCCO	-189.33	
	1.00		12	14	15	16	CCNC	-103.32	
s147	1.00	OUT	12	14	11	36	CCCH	23.64	f1459 13 f1420 17 f1372 11 f1299 17
s148	1.00	OUT	13	11	14	12	OCCC	57.30	
s149	1.00	OUT	18	14	16	15	CCCN	41.55	f117 24
	-1.00	TORS	15	16	19	20	NCCO	-189.33	
s150	1.00	OUT	17	19	15	16	CCNC	46.71	f1278 10 f543 21 f447 11 f398 18
s151	1.00	OUT	9	8	1	7	CCCC	50.82	f590 11
s152	-1.00	OUT	10	3	5	4	OCCC	0.06	f570 50
	1.00		7	2	6	1	CCCC	0.03	
s153	1.00	OUT	7	2	6	1	CCCC	0.03	f781 28 f434 15 f248 11
	1.00		10	3	5	4	OCCC	0.06	

****

```
52 STRE modes:
 1  2  3  4  5  6  7  8  9 10 11 12 13 14 15 16 17 18 19 20
21 22 23 24 25 26 27 28 29 30 31 32 33 34 35 36 37 38 39 40
41 42 43 44 45 46 47 48 49 50 51 52

51 BEND modes:
53 54 55 56 57 58 59 60 61 62 63 64 65 66 67 68 69 70 71 72
73 74 75 76 77 78 79 80 81 82 83 84 85 86 87 88 89 90 91 92
93 94 95 96 97 98 99 100 101 102 103

50 TORS modes:
104 105 106 107 108 109 110 111 112 113 114 115 116 117 118 119 120 121 122 123
124 125 126 127 128 129 130 131 132 133 134 135 136 137 138 139 140 141 142 143
144 145 146 147 148 149 150 151 152 153

90 CH modes:
 2  3  4  5  6  7  8  9 10 11 12 13 14 15 16 17 18 19 20 21
22 23 24 25 26 27 28 29 30 31 55 56 57 58 59 60 61 62 63 64
65 66 67 68 69 70 71 72 73 74 75 76 77 78 79 80 81 82 83 84
105 106 107 108 109 110 111 112 113 114 115 116 117 118 119 120 121 122 123 124
125 126 127 128 129 130 131 132 133 147
```

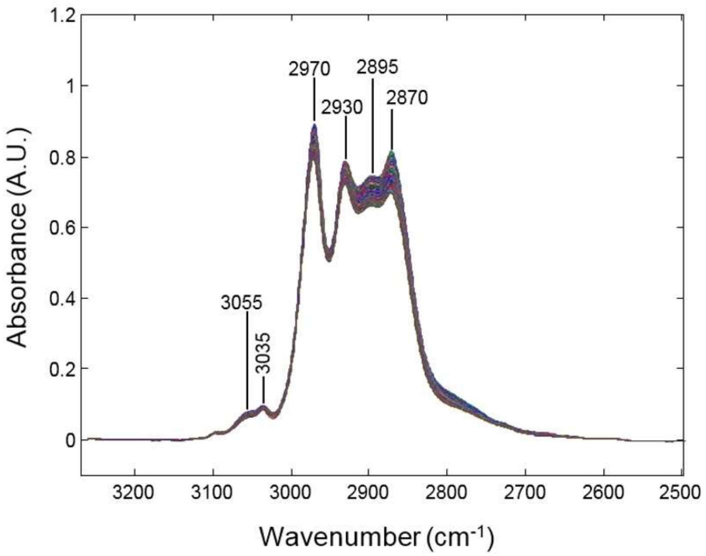

Figure S4. *Full sequence of spectra in the 3200 – 2500 cm^{-1} range employed for the 2D-COS analysis.*

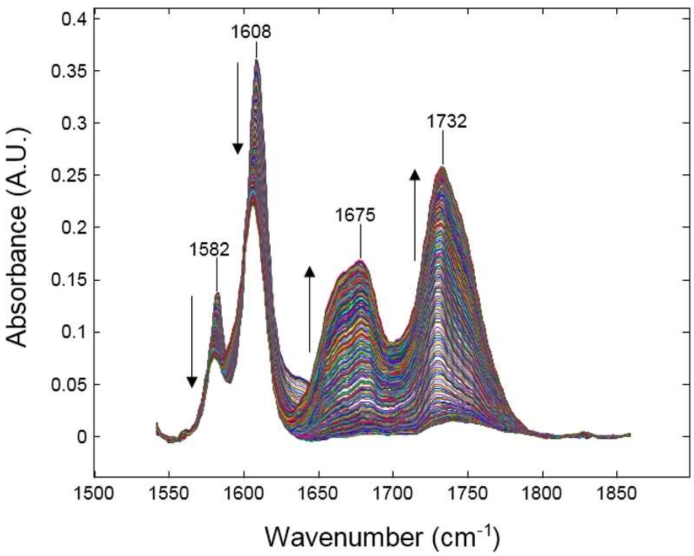

Figure S5. *Full sequence of spectra in the 1500 – 1900 cm^{-1} range employed for the 2D-COS analysis.*
